# Mapping the major B-cell linear epitope of silkworm (*Bombyx mori*) pupal chitinase and molecular dynamics insights into its IgE-binding stability

**DOI:** 10.3389/fimmu.2026.1942595

**Published:** 2026-10-01

**Authors:** Lei Gu, Xiangjie Zhao, Lei Yan, Lin Bo, Xuemei Sui

**Affiliations:** 1 The Affiliated Huai’an No. 1 People’s Hospital of Nanjing Medical University, Huai’an, China; 2 Huai’an University, School of Life Science and Food Engineering, Huai’an, China; 3 The Second Affiliated Hospital of Soochow University, Suzhou, China

**Keywords:** allergen chitinase, epitopes, molecular dynamics simulation, overlapping peptide, silkworm (*Bombyx mori*) pupae

## Abstract

As domestic silkworm (*Bombyx mori*) pupae emerge as a promising alternative food source, the severe IgE-mediated allergic reactions they trigger have raised significant public health concerns. Despite the identification of the novel allergen *Bombyx mori* chitinase (BmChi), its precise linear B-cell epitopes and molecular mechanisms of IgE recognition remain uncharacterized. In this hypothesis-generating study, we systematically mapped the linear B-cell epitopes of BmChi and evaluated their structural dynamics. Following bioinformatic predictions, a high-resolution *in vitro* screening using 109 overlapping peptides successfully identified peptide S31 (residues 536-548) as the major dominant epitope. To gain atomistic insights into the BmChi-IgE interaction, homology modeling and molecular docking were conducted, revealing a structurally stable complex supported by extensive hydrogen-bonding networks within flexible loop regions. Furthermore, molecular dynamics simulations (MDs) and steered molecular dynamics simulations (SMDs) provided dynamic evidence of the complex’s remarkable rigidity and mechanical resistance. Additionally, phylogenetic analysis highlighted the conserved nature of BmChi relative to plant panallergens. These findings should be interpreted as computational predictions corroborated by *in vitro* peptide-level binding data rather than direct clinical evidence of allergenic potency. The predicted consensus epitopes and their elucidated structural robustness provide a precise molecular basis for mitigating insect-derived allergenic risks through directed food processing and immunotherapy design.

## 1 Introduction

As global demand for sustainable and highly nutritious alternative proteins continues to rise, driven by the need to ensure food security and mitigating environmental degradation, edible insects, particularly the pupae of the domestic silkworm (*Bombyx mori*), have emerged as a promising resource owing to their exceptional nutritional profile and historical medicinal applications (1, 2). However, large-scale industrialization and dietary incorporation of insect-based foods are significantly impeded by the escalating incidence of IgE-mediated food allergies (3, 4). Allergic reactions to edible insects can trigger severe, life-threatening anaphylaxis, particularly in susceptible individuals with pre-existing sensitization to phylogenetically related arthropods such as crustaceans or house dust mites (5, 6). Effective clinical management of these food allergies currently relies on strict dietary avoidance and precise molecular diagnostics, which inherently necessitate the comprehensive identification of clinically relevant allergenic proteins (7). While major silkworm allergens such as arginine kinase, tropomyosin, and the 30 kDa lipoprotein have been characterized, the broader spectrum of allergenic determinants in *B. mori* remains incompletely elucidated, thereby restricting the advancement of targeted immunotherapies and hypoallergenic food processing (8, 9).

Allergic sensitization and the subsequent elicitation of immune cascades are fundamentally driven by the interaction between specific IgE antibodies and discrete structural moieties on the allergen surface, known as antigenic epitopes (10, 11). These epitopes, whether linear (continuous amino acid sequences) or conformational (discontinuous patches assembled through three-dimensional folding), survive gastrointestinal degradation to cross-link IgE receptors on effector cells (12). Consequently, precise epitope mapping is critical for understanding allergenic mechanisms and for guiding site-directed hypoallergenic modifications (13, 14). In this context, chitinase (EC 3.2.1.14), a ubiquitous enzyme pivotal for chitin degradation and pathogen resistance, has recently garnered significant attention. Historically recognized as a major class of plant-derived panallergens (e.g., in latex, kiwifruit, and banana) responsible for the latex-fruit cross-reactive syndrome, the pedigree of chitinase allergens has now expanded to edible insects (15, 16). Notably, a novel chitinase allergen from silkworm pupae has been identified, exhibiting considerable sequence homology with established dust mite allergens (e.g., Der f 18 and Der p 18) (17). This striking evolutionary conservation underscores an urgent need to evaluate the cross-reactive potential and structural immunogenicity of insect-derived chitinases.

Despite its clinical relevance, the specific IgE-binding epitopes of BmChi remain largely uncharacterized. While traditional *in vitro* techniques, such as overlapping peptide synthesis and enzyme-linked immunosorbent assay (ELISA), are highly effective for mapping linear epitopes, they fundamentally fail to capture complex conformational epitopes that represent the vast majority of native IgE-binding sites (18). These discontinuous epitopes are highly dynamic and intricately dependent on the overall spatial architecture of the protein, rendering them highly susceptible to physical processing and environmental factors (12, 19). To overcome these technical bottlenecks, modern structural allergology increasingly relies on advanced immunoinformatics and *in silico* modeling. Techniques such as molecular docking provide essential static snapshots of antigen-antibody interfaces, whereas MDs and SMDs offer profound atomistic insights into the kinetic stability, conformational flexibility, and mechanical unbinding pathways of allergen-IgE complexes (20–22). These computational tools allow researchers to transcend traditional equilibrium timescales, directly observing how external forces and solvent environments impact epitope-paratope engagement (23).

To bridge this critical knowledge gap, the present study proposes an integrated methodology combining empirical *in vitro* validation with advanced *in silico* simulations to comprehensively characterize the allergenic profile of BmChi. The primary objective was to map the linear B-cell IgE-binding epitopes of BmChi experimentally and to identify the immunodominant linear epitope, using an unbiased 109-peptide overlapping screen and peptide-ELISA with anti-BmChi IgE. The secondary objectives were to reconstruct the BmChi-IgE complex by homology modeling and molecular docking, to characterize its conformational and mechanical stability using MDs and SMDs, and to position BmChi within the broader chitinase-allergen family while evaluating its predicted cross-reactive potential. On this basis, and motivated by the observation that structurally conserved regions in panallergens can act as IgE-binding triggers, we prespecified the hypothesis that BmChi contains one or more immunodominant linear B-cell epitopes situated within evolutionarily conserved, surface-accessible loops, and that these epitopes interact with IgE via a stable, hydrogen bond-stabilized interface.

Initially, bioinformatic algorithms were employed to predict potential B-cell linear epitopes, which were subsequently validated through the synthesis of overlapping peptides and immune screening via ELISA using specific antisera. Furthermore, to capture the native non-linear interactions, high-fidelity homology modeling and molecular docking were conducted to reconstruct the three-dimensional complex of BmChi and IgE-Fv. Crucially, extensive MDs and SMDs were executed to elucidate the dynamic binding kinetics, interface rigidity, and mechanical dissociation pathways of the complex. By mapping the dominant linear epitopes and verifying the robust spatial engagement of conformational sites, this study aims to unravel the molecular basis of BmChi’s potent allergenicity. Ultimately, deciphering these atomistic interactions will provide a vital theoretical foundation for the development of precise molecular diagnostics and targeted food processing strategies to mitigate the allergenic risks of insect-derived alternative proteins.

## 2 Materials and methods

This is a hypothesis-generating, laboratory-based study that integrates empirical *in vitro* validation with *in silico* structural simulation. The target protein was recombinant BmChi from domestic silkworm (*Bombyx mori*) pupae. Anti-BmChi IgE antiserum was generated in mice immunized with the recombinant protein; no human subjects or clinical samples were involved. The integrated workflow commenced with bioinformatic prediction of linear B-cell epitopes, followed by synthesis of 109 overlapping peptides and peptide-based ELISA screening. Subsequent steps included homology modeling and molecular docking of the BmChi-IgE complex, and culminated in MDs and SMDs to assess complex stability. The complete experimental procedure is schematically summarized in **Scheme 1**.

### 2.1 Materials

Silkworm (*Bombyx mori* L.) pupae were donated by the Sericultural and Agro-Food Research Institute, Guangdong Academy of Agricultural Sciences, and stored at -78 °C in liquid nitrogen until used. The avidin HRP (Horseradish peroxidase) and TMB (3,3’,5,5’-Tetramethylbenzidine) solutions were purchased from eBioscience (USA). Biotin-labeled affinity-purified antibody to mouse IgE and streptavidin were obtained from KPL (Gaithersburg, MD, USA). All other analytical reagents were purchased from Sigma (USA).

Total RNA was extracted from silkworm pupae using the TRIzol^®^ method, The full-length cDNA of silkworm pupal chitinase (BmChi) was amplified by reverse transcription polymerase chain reaction (RT-PCR), the upstream and downstream primers are as follows: 5’GGCGATCTGGTGACTTATGATGA3’ and 5’GCGGAATTTCCTAAGCCCATATTC3’. The recombinant chitinase protein expressed in a heterologous system following genomic cloning and subsequent induction was supplied by Ruizhen Bio-Technology Company (Guangzhou, Guangdong, China). The antibody serum prepared with recombinant chitinase protein was provided by Zvast-Bio Company (Nanchang, Jiangxi, China). Detailed information on the RT-PCR primers, genomic cloning, expression and induction conditions is provided in the Supplementary Methods. There was no human *in vivo* experimentation, because BmChi is a recently identified novel allergen for which no clinically characterized human allergic sera with documented silkworm-chitinase sensitization are available in our setting, mouse IgE was used as a validated surrogate for screening linear-peptide epitopes.

### 2.2 Synthesis of BmChi overlapping antigen epitope

Linear overlapping peptide segments (15-mer peptides with a 5-amino acid overlap) spanning the entire BmChi sequence were synthesized by Shanghai ZYBio Company (China) using Fmoc solid-phase peptide synthesis on a Vantage fully automated synthesizer (AAPPTEC, USA). The overlapping-peptide and alanine-scanning design follows the experimental paradigm reported in previous work (24); both that study and the present work employ the same Fmoc-based solid-phase peptide synthesis strategy, however, the specific synthesis provider and instrument differed. A total of 109 overlapping peptides were synthesized; their sequences are listed in **Supplementary Table S1**. The purity of the synthesized 15 peptide segments exceeded 95%. Purity analysis was carried out via high-performance liquid chromatography (HPLC) using an Agilent 1100 system with a C18 reverse-phase column, gradient elution using acetonitrile/water with 0.1% TFA, and detection at 214 nm. Peptide molecular weights were confirmed by ESI-MS on a Waters ZQ2000 instrument. Then, the synthesized peptides were stored at -20 °C for use. For highly reactive peptides identified via peptide antigenicity by ELISA, peptide sequence redesign was performed. Starting from the original 15-mer peptides, amino acid residues were systematically truncated (either from the N-terminus or the C-terminus) one residue at a time, until the length was reduced to 10 residues. Furthermore, the contribution of individual amino acid residues to antigenicity was assessed using alanine-scanning mutagenesis, wherein each residue in the peptide segment was substituted with alanine (Ala) in a stepwise manner. Detailed structural characterizations of the synthesized peptides are provided in **Supplementary Table S2**.

### 2.3 Sequence retrieval and homology analysis

Chitinase is a key enzyme in the chitin degradation metabolic pathway. It is widely distributed across diverse biological taxa and has been identified in insects, crustaceans, bacteria, fungi, and other organisms. Its functional roles include immunology, allergic responses, nutrition, and morphogenesis (15, 25). The full-length amino acid sequence of BmChi (accession number: Q869E2) was retrieved from the UniProt protein database (https://www.uniprot.org). Sixteen chitinase sequences from diverse biological sources were obtained via protein to protein BLAST (BLASTP) searches against the National Center for Biotechnology Information (NCBI) non-redundant protein database. Sequence similarity was evaluated with BLASTP (NCBI BLAST+) against the non-redundant protein database, using the BmChi sequence as a query with an expectation (E) value threshold of ≤1×10^-5^; compositional adjustment of substitution matrices (CASM) was applied to improve the accuracy of biased-composition regions (26). To capture more distant homologs, position-specific iterated BLAST (PSI-BLAST) was additionally performed, which reduces false negatives by constructing a position-specific scoring matrix from the initial hits (27). Alignment coverage (the fraction of the query length aligned to each hit) was recorded for every retrieved sequence. Two authoritative online allergen databases, AllergenOnline (http://www.allergenonline.org/) (28) and Allergome (https://www.allergome.org) (29), were used to identify known chitinase allergens. A representative set of 23 characterized chitinase allergens, including BmChi, were subjected to multiple sequence alignment and phylogenetic tree reconstruction using BioEdit and ClustalW for a direct homology analysis.

### 2.4 Comprehensive prediction of B-cell linear epitopes for BmChi

To systematically predict the B-cell linear epitopes of BmChi, the Protean module within the DNASTAR software suite was employed, focusing on four core physicochemical properties of the amino acid sequence: hydrophobicity and hydrophilicity, flexibility, antigenicity, and surface probability. Specifically, hydrophobicity and hydrophilicity were evaluated using the Hopp-Woods methods. Sequence flexibility, antigenic index, and surface probability were assessed using the Karplus-Schulz, Jameson-Wolf, and Emini methods (30), respectively. Additionally, the Bioinformatics Predicted system (BepiPred 2.0) server (https://services.healthtech.dtu.dk/services/BepiPred-2.0) was utilized to corroborate the linear epitope predictions. Biologically, regions characterized by high hydrophilicity, pronounced flexibility, and surface accessibility are structurally more predisposed to interact with antibodies (31). Therefore, continuous stretches of amino acid residues with physicochemical scores meeting or exceeding the established threshold values were designated as potential dominant linear epitopes and extracted for downstream validation.

### 2.5 Assessment of the antigenicity of BmChi allergic epitopes

Antigenic epitopes are discrete, structurally defined chemical moieties that confer antigenicity to allergenic proteins. Currently, computational prediction methods are widely employed to identify key regions capable of binding specific antibodies by integrating amino acid sequence data, physicochemical properties, and three-dimensional structural features (32). To comprehensively map the theoretical linear epitopes of BmChi, we evaluated multiple critical physicochemical parameters: hydrophilicity, hydrophobicity, flexibility, surface accessibility, and antigenicity index. The antigenicity of the antigenic epitopes of BmChi overlapping peptides was assessed via the ELISA method (33). Briefly, dissolved overlapping peptides were serially diluted in PBS buffer to yield final peptide concentrations of 2.50 μg/μL. In an amino acid-binding 96-well plate (Thermo, USA), 100 μL of PBS buffer and 2 μL of the appropriately diluted peptide sample were added to each well. Plates were sealed with parafilm and incubated overnight at 4 °C on a microplate shaker set to gentle agitation. Following incubation, the plate was removed from the cold chamber and equilibrated to room temperature for 15 minutes. Prepared mouse IgE serum was diluted at a 1:1000 ratio, and then mixed with an equal volume of overlapping peptide and preincubated for 1 h. The coating solution was then aspirated and discarded, after washing each well, 100 μL of blocking solution was added and incubated for 1 hour at room temperature. After washing, 100 μL of primary antibody working solution and 100 μL of biotin-conjugated antibody were added separately and incubated for 1 hour at room temperature. After washing, 100 μL of HRP-conjugated streptavidin working solution was added to each well and incubated for 1 hour at room temperature. Subsequently, 100 μL of 1× TMB substrate solution was rapidly added to each well and incubated for 10 minutes at room temperature in the dark. The enzymatic reaction was terminated by promptly adding 100 μL of 2 M sulfuric acid (stop solution) to each well. Finally, absorbance was measured at 450 nm using a microplate reader. All ELISA experiments were performed in triplicate (three independent biological replicates), with each replicate measured in duplicate technical repeats. Positive control: mouse anti-BmChi polyclonal serum; Negative control: PBS buffer. The cutoff for a positive peptide was defined as OD450 ≥ 0.30, which represents the mean OD of all negative control wells plus three times the standard deviation.

### 2.6 Homology modeling and molecular docking

Molecular docking technology holds significant potential as an *in silico* screening tool to predict the interaction between potential allergenic epitopes and specific immune receptors (34). The binding affinity between the allergenic peptides and their corresponding protein targets dictates the docking scores of the candidates, providing a reliable and quantitative basis for the identification and screening of dominant epitopes (33). The 3D structure of BmChi was constructed via homology modeling using MODELLER 9, based on sequence alignment (DIALIGN) with *Serratia marcescens* chitinase templates (PDB ID: 1RD6, 2WK2, and 2WLY). A template with a relatively high structural homology score was selected for model construction in the following experiment. The Chimera software (http://www.cgl.ucsf.edu/chimera/) was employed to remove small molecules, ligands, water molecules, and other components that aid in protein crystallization. The constructed model was energy-minimized using YASARA V13 with the AMBER99 force field and evaluated by PROCHECK and Verify_3D. For molecular docking, the crystal structure of the IgE (PDB ID: 1OAZ) fragment of antigen binding was obtained from the PDB database. Protein-protein flexible docking between the BmChi model and the IgE protein was simulated using ZDOCK (35). Poses with a ZDOCK score above 12 were refined and rescored using RDOCK (36). The binding modes and intermolecular interactions were analyzed and visualized using PyMOL. Because structural congruence can be more conserved than primary sequence among allergen B-cell epitopes, the modeled BmChi fold and its key interfacial loops were additionally validated by structural superposition; this confirmed that the predicted epitope-bearing regions adopt a fold consistent with canonical chitinases (37).

### 2.7 Dynamics

To evaluate the conformational stability of the BmChi-IgE complex in a simulated aqueous environment, a 20 ns MDs was performed. The accurate modeling of such initial docked conformations and their subsequent dynamic trajectories has become a cornerstone in modern structural biology for evaluating protein interactions (38). To gain deeper insight into the binding mechanism between BmChi and the Fv fragment of mouse IgE antibodies, MDS of the BmChi-IgE complex was performed using the YASARA V13 software package and the AMBER99 force field. The complex was solvated in a periodic water box with a 10 Å buffer and neutralized by Na^+^ ions. The simulations were carried out with the following parameters: integration time step of 1 fs, temperature set to 300 K, and pressure set to 1 atm. Long-range electrostatic interactions were calculated using the PME method. Following a 5000-step steepest descent minimization and an 80 ps position-restrained equilibration, a single 20 ns conventional MDS was performed, and the trajectory convergence was confirmed by a backbone RMSD plateau reached after ~4 ns and by interfacial RMSF values < 1 Å, indicating that 20 ns provided adequate sampling of the bound-state ensemble to assess complex stability. Subsequently, a 250 ps SMDs was conducted to investigate the unbinding process. An external force was applied to the IgE atoms to pull it out of the complex along defined pathways, with the optimal pulling velocity set to 0.032 Å/ps. Thus, the system was subjected to two dynamics simultaneously: one is a conventional MDS for 20 ns, and the other is an SMDs for 250 ps.

### 2.8 Statistical analysis

All experiments were repeated at least three times, and the antigenicity of synthetic allergic epitopes was presented as the mean ± standard deviation (SD) with Origin 2024 (OriginLab, Massachusetts, USA). The MDs data were analyzed using one-way ANOVA with Duncan’s multiple range test with the SPSS 20 software (SPSS Inc., Chicago, USA) at a statistically significant level of p-value < 0.05. All values that are repeated at least three times in the supplementary file. All p-values are two-tailed, and findings with p ≥ 0.05 are explicitly reported as ‘not statistically significant’.

## 3 Results

### 3.1 Homology analysis of BmChi with diverse chitinases and known allergens

To elucidate the evolutionary relationships of BmChi, a phylogenetic tree was constructed using 16 chitinase sequences from diverse taxa. Phylogenetic analysis segregated these chitinases into two primary clades (**Figure 1A**). BmChi robustly clustered within the first clade, exhibiting high sequence homology with chitinases from closely related lepidopterans (*Manduca sexta*, *Agrius convolvuli*, *Samia cynthia*, and *Antheraea pernyi*). The second major clade further diverged into two distinct lineages encompassing other insect-derived chitinases (e.g., *Spodoptera litura*, *Spodoptera exigua*, *Mythimna separata*, *Mamestra brassicae*, *Ostrinia furnacalis*, *Papilio xuthus*, *Papilio polytes*, and *Danaus plexippus*) and bacterial chitinases (*Serratia grimesii*, *Serratia liquefaciens*, and *Serratia marcescens*).

**Figure 1:**
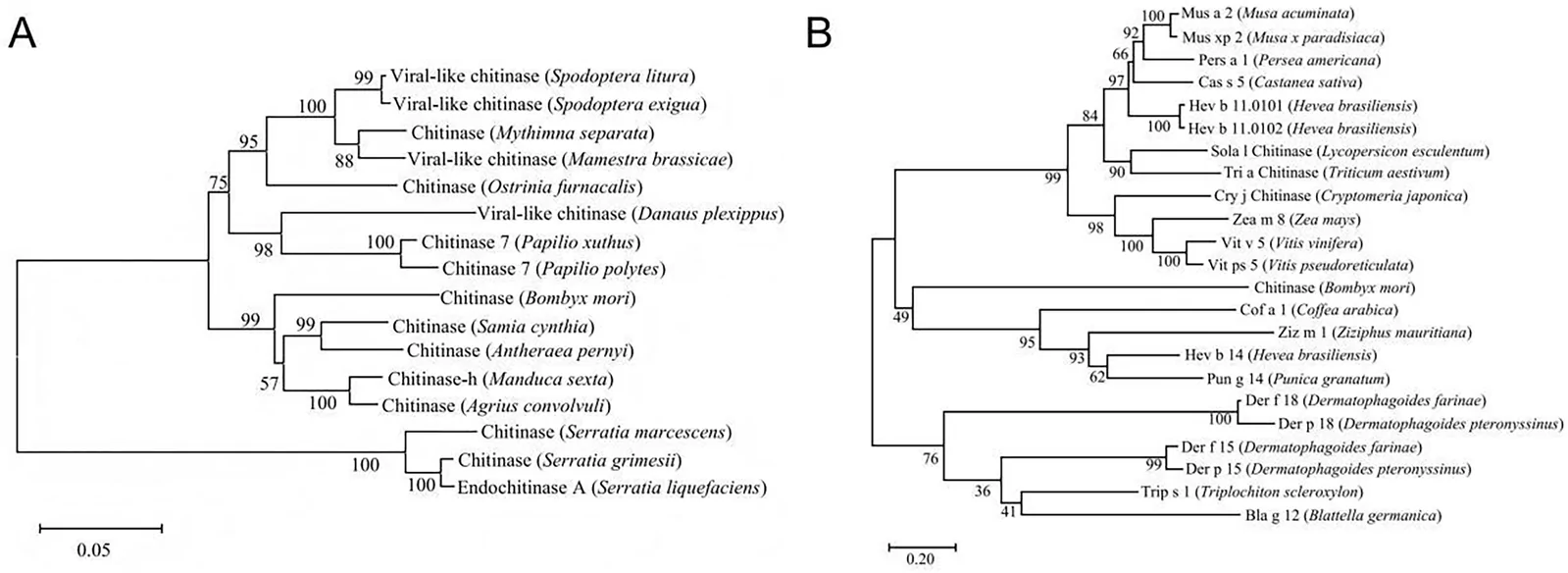
Phylogenetic relationships of BmChi (*Bombyx mori*, domestic silkworm) with functionally and immunologically related chitinases retrieved from the UniProt protein knowledgebase. **(A)** Phylogram of BmChi and chitinases from diverse species; **(B)** phylogram of BmChi and characterized chitinase allergens. Amino acid sequences were aligned using ClustalW as implemented in BioEdit. Phylogenetic trees were reconstructed by the neighbor-joining (NJ) method; branch lengths are proportional to the number of amino-acid substitutions per site. Branch robustness was assessed with 1,000 bootstrap replicates, and bootstrap support values (%) are shown at each node. The scale bar represents amino-acid substitutions per site.

Subsequent queries of the Allergen and Allergome databases revealed a lack of documented chitinase allergens from BmChi-related species (e.g., Manduca *sexta*, *Agrius convolvuli*). Consequently, a secondary phylogenetic tree was generated utilizing 22 known chitinase allergens (**Figure 1B**). Strikingly, BmChi displayed high sequence similarity with several well-characterized plant-derived allergens, notably Cof a 1 (*Coffea arabica*), Hev b 14 (*Hevea brasiliensis*), Ziz m 1 (*Ziziphus mauritiana*), and Pun g 14 (*Punica granatum*). The pairwise sequence identity between BmChi and these plant chitinase allergens ranged from 50% to 72%, with the highest similarity observed for Ziz m 1 (72%).

### 3.2 Prediction and characterization of BmChi epitopes

Optimal candidates were filtered using established theoretical thresholds (hydrophilicity > 0, flexibility > 1, surface accessibility > 1, and antigenicity index > 0) and subsequently benchmarked against the predictive algorithms of BepiPred and Kolaskar & Tongaonkar (**Figures 2A–F**) (39). Although spatially dispersed across the primary sequence, the predicted sites predominantly clustered within four major regions (residues 15-80, 110-235, 320-430, and 490-550). Notably, these segments exhibit high sequence conservation with homologous insect and mite allergens (e.g., Der p 18, Der f 18, Der p 15, and Der f 15) (17), strongly indicating the presence of viable, evolutionarily conserved IgE-binding sites. Structurally, these highly accessible and hydrophilic segments are typically 8–20 amino acids in length, predominantly form flexible random coils, and are significantly enriched in charged polar residues (such as arginine, tyrosine, and tryptophan) while largely lacking valine. Based on this multiparametric consensus analysis, 12 final linear epitope sites were pinpointed: 23-37, 66-74, 70-82, 107-120, 139-153, 192-201, 228-236, 270-279, 308-327, 402-415, 497-510, and 537-555. Notably, the predicted epitope P12 (residues 537-555) is located adjacent to the catalytically active site of the chitinase (Asp306, Asp308, Glu310), within the functionally conserved (β/α)_8_-barrel core domain, and encompasses Trp539, a residue previously identified as critical for substrate binding (15). In summary, the multi-parameter forecasting strategy adopted in this study is both well-justified and scientifically rigorous.

**Figure 2:**
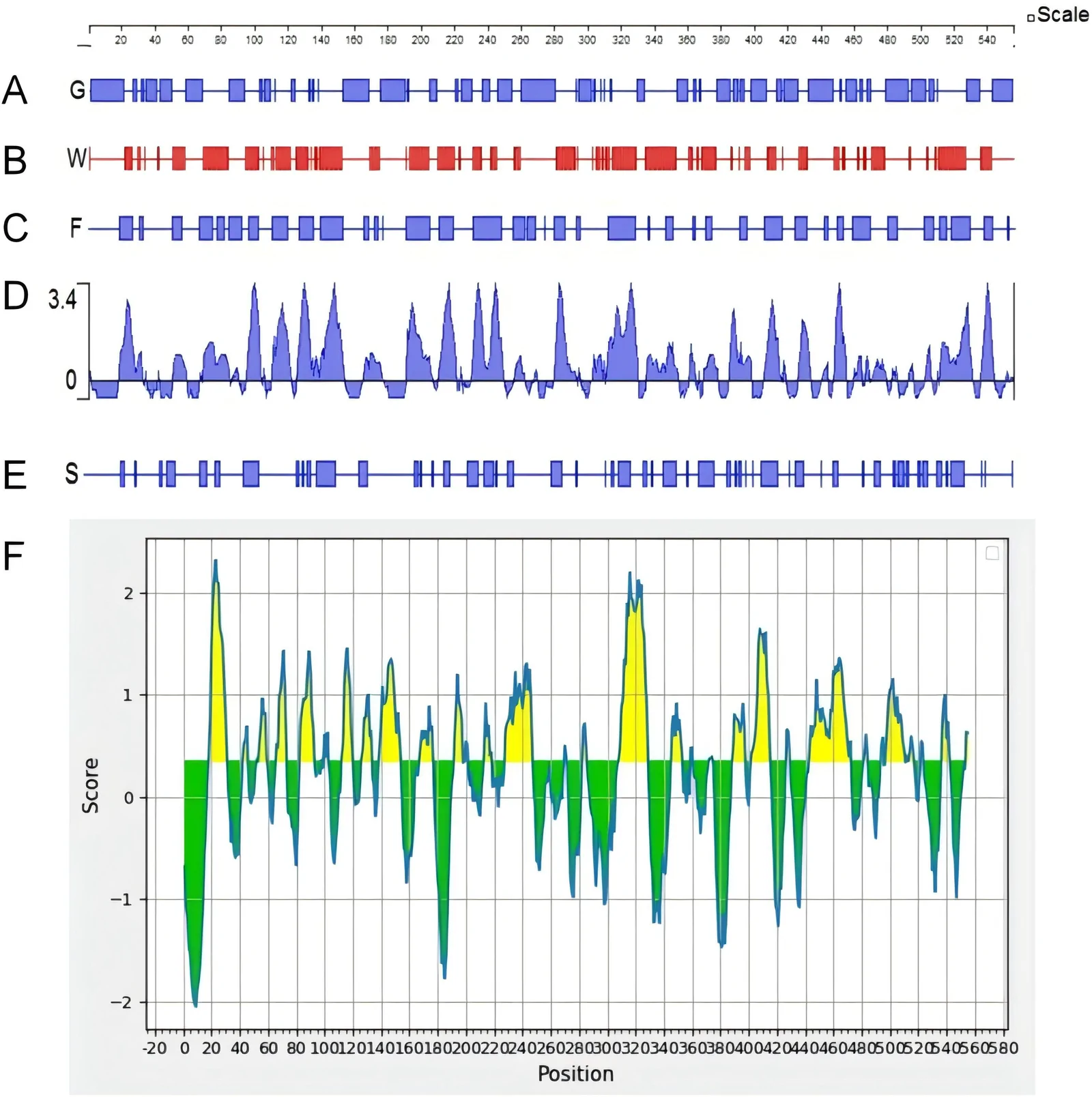
Physicochemical profiling and linear B-cell epitope prediction of BmChi. All profiles were generated with the Protean module of the DNASTAR Lasergene suite. **(A)** Hydrophobic regions and **(B)** hydrophilic regions, computed with the Hopp-Woods scale; **(C)** flexible regions (Karplus-Schulz flexibility); **(D)** antigenic index (Jameson-Wolf); **(E)** surface-probability regions (Emini). **(F)** B-cell epitope prediction by the BepiPred 2.0 server, corroborated with the Kolaskar & Tongaonkar antigenicity scale.

### 3.3 Antigenicity validation of synthetic allergic epitopes

To experimentally map the B-cell linear epitopes of BmChi and validate the *in silico* predictions, a library of 109 overlapping peptides (15-mers with a 5-amino-acid offset) spanning the entire protein sequence was synthesized and comprehensively evaluated via an ELISA test. While the majority of peptides exhibited baseline reactivity (OD < 0.30), peptides P24 and P108 demonstrated highly significant specific binding capacities, yielding peak absorbance values of 0.93 and 1.01, respectively (**Figure 3A**). To precisely pinpoint the core binding sequences, these two highly reactive candidates were subjected to fine mapping via terminal truncation (sequentially reduced to 10-mers) and alanine scanning mutagenesis.

**Figure 3:**
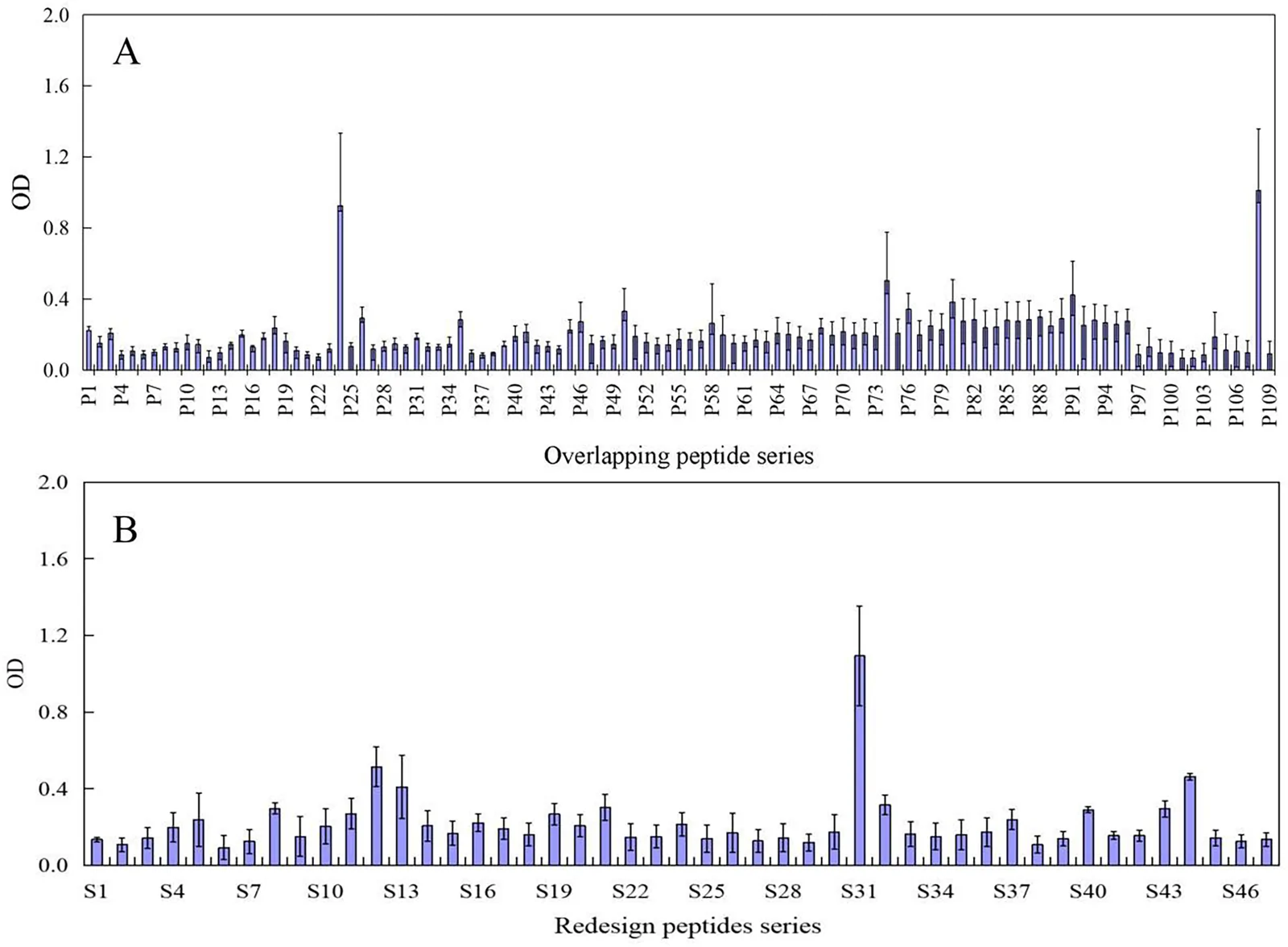
Antigenic profiling of BmChi-derived peptides by indirect peptide-ELISA. **(A)** Antigenic reactivity of a library of 109 overlapping 15-mer peptides spanning the full-length BmChi sequence (residues 1-550; 5-aa offset). Reactivity was measured by indirect peptide-ELISA using mouse anti-BmChi IgE sera; the mean OD450 (± SD) is plotted on the y-axis against the N-terminal residue position of each peptide on the x-axis. Full details of all 109 peptides are provided in **Supplementary Table S1**. **(B)** Antigenicity of truncated and redesigned derivatives of the immunodominant peptides P24 and P108, generated by systematic N-/C-terminal truncation and alanine-scanning mutagenesis. Full peptide sequences, residue positions, and synthesis details are provided in **Supplementary Table S2**.

Subsequent ELISA evaluation of these refined variants (**Figure 3B**) revealed that derivative peptide S31 (BmChi residues 536-548, sequence: IDADN GDILN AMN, a C-terminally truncated derivative of P108, which spans residues 536-550) exhibited the most robust binding affinity, achieving an exceptional peak absorbance of 1.093. Conversely, related derivatives (e.g., S12, S13, and S44) displayed substantially reduced reactivity (OD 0.410-0.515). This step-wise experimental validation identifies S31, corresponding to residues 536–548 of the BmChi allergen, as the dominant linear epitope among the sequences screened, indicating its critical role in mediating specific allergic reactivity, with clinical allergenicity still to be confirmed in human sera. These empirical findings perfectly corroborate our computational predictions, underscoring the reliability of integrating sequence-derived features with antigenicity models, and highlighting specific amino acid residues within the S31 sequence as the central determinants of antibody-binding.

### 3.4 Molecular docking analysis of BmChi and IgE

A three-dimensional homology model of the BmChi protein was constructed using MODELLER, followed by structural optimization and refinement (**Figure 4A**). Consistent with the template chitinase, the BmChi structure comprises three distinct domains, featuring a central (β/α)_8_-barrel core composed of eight parallel β-strands surrounded by eight α-helices. These helices are connected to the barrel core via linkers of varying lengths and conformations. Overall, the structure contains 12 α-helices and 25 β-turns, which are characteristic structural motifs commonly observed in chitinases (25).

**Figure 4:**
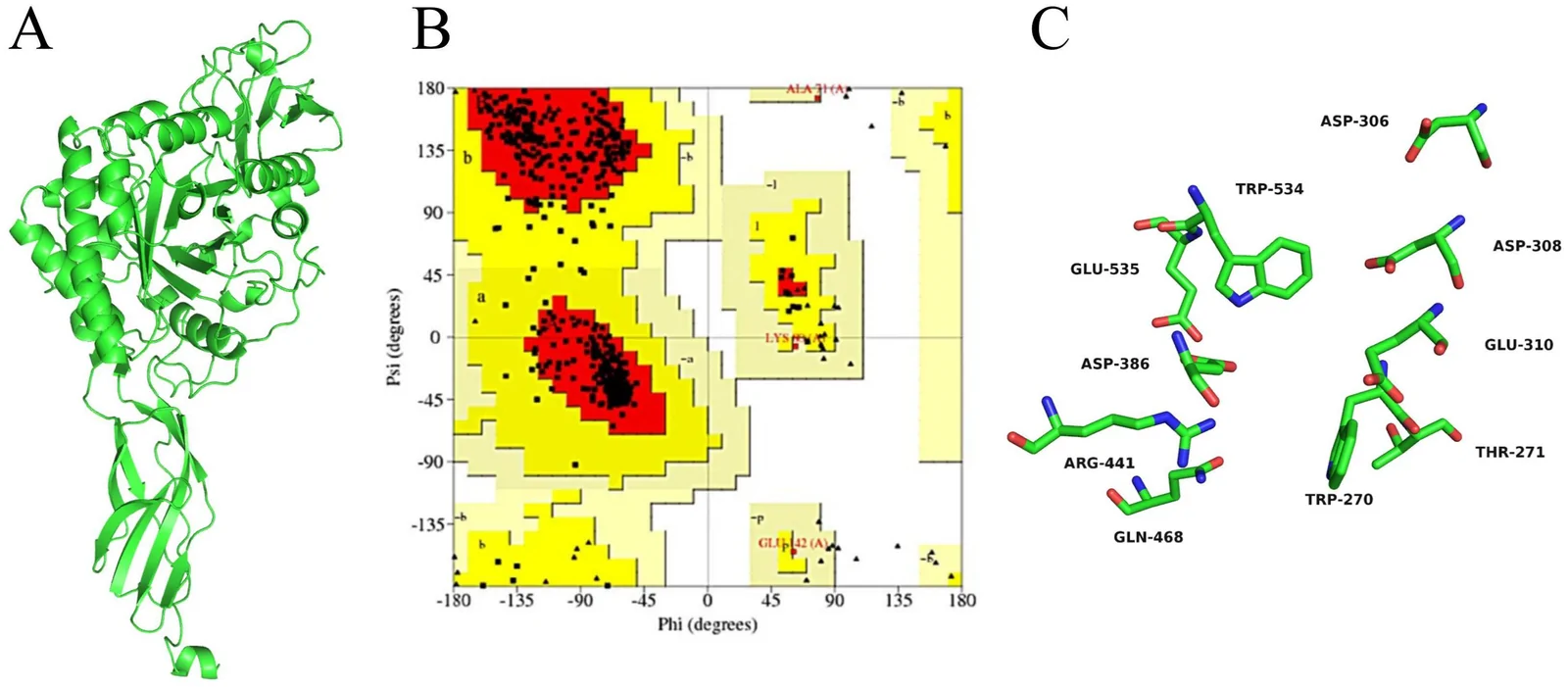
Homology-modeled 3D structure of BmChi and its active-site architecture. Sequence alignment results of BmChi with high homology chitinase templates are listed in **Supplementary Table S3**. **(A)** 3D structure of BmChi; **(B)** Ramachandran plot of the modeled structure, generated with PROCHECK, showing the distribution of φ/ψ backbone dihedral angles (favored/allowed regions: %); **(C)** key catalytic and substrate-binding residues lining the active-site pocket.

To ensure structural validity, the stereochemical quality of the optimized BmChi model was evaluated using PROCHECK (40) and Verify 3D (**Supplementary Figure S1**). The Ramachandran plot analysis was performed to assess the structural reliability and stability of the model (41). As shown in **Figure 4B**, the plot revealed that 90.6% of the amino acid residues were positioned in the most favored regions, 8.7% in additionally allowed regions, 0.7% in generously allowed regions, and 0.0% in disallowed regions. With 99.3% of the residues located within acceptable regions, these results confirm the high stereochemical reliability and structural plausibility of the BmChi model, making it highly suitable for subsequent active site analysis and molecular docking studies.

Based on the conserved catalytic pocket of canonical chitinases, the putative ligand-binding site of BmChi was predicted, identifying key catalytic and substrate-interacting residues (**Figure 4C**). Specifically, Asp306, Asp308, and Glu310 are positioned to form hydrogen bonds with small-molecule ligands, while Trp275 and Trp539 facilitate hydrophobic interactions within the active site (15), which is highly consistent with the substrate-binding cleft architecture previously reported for *Bombyx mori* chitinase (42). Notably, the conformation of Glu310 in BmChi differs markedly from its corresponding residue in the homologous template. This conformational divergence may influence the binding orientation and pose of ligands within the active pocket, potentially driving differences in enzymatic selectivity and substrate specificity. Nevertheless, the spatial arrangement and side-chain conformations of other key active-site residues in BmChi exhibit high fidelity to the template. This underscores the strong structural homology and evolutionary conservation of the functional domains, an important structural aspect when evaluating the potential cross-reactivity and allergenicity of silkworm pupae in food safety (2), further supporting the accuracy of the constructed model.

Through structural analysis of the BmChi-IgE binding complex, the docking model indicates that the IgE-Fv fragment binds robustly to the surface of the BmChi protein (**Figure 5A**). The three-dimensional architecture of the complex reveals high shape complementarity between the IgE-Fv fragment and the BmChi binding interface. Specifically, the turn region of the IgE-Fv inserts deeply into the base of the BmChi catalytic cavity. Furthermore, Loops 1, 2, and 3 collectively engage the BmChi surface through extensive intermolecular contacts. Notably, Loop 1 forms particularly strong interactions with the concave region of BmChi. Concurrently, the β-sheet of IgE-Fv aligns parallel to this concave surface. This precise geometric arrangement significantly minimizes steric clashes arising from structural mismatches, thereby facilitating a stable, high-affinity interaction between the antibody fragment and the allergen (43). The interaction of the BmChi-IgE complex is mediated by five key structural elements: the Turn, Loop 1, Loop 2, Loop 3, and the β-sheet, corresponding to the binding-region frameworks illustrated in **Figures 5B**, **C**. This binding mode structurally resembles a “five-claw” configuration, enabling a stable, multivalent grasp of the BmChi surface.

**Figure 5:**
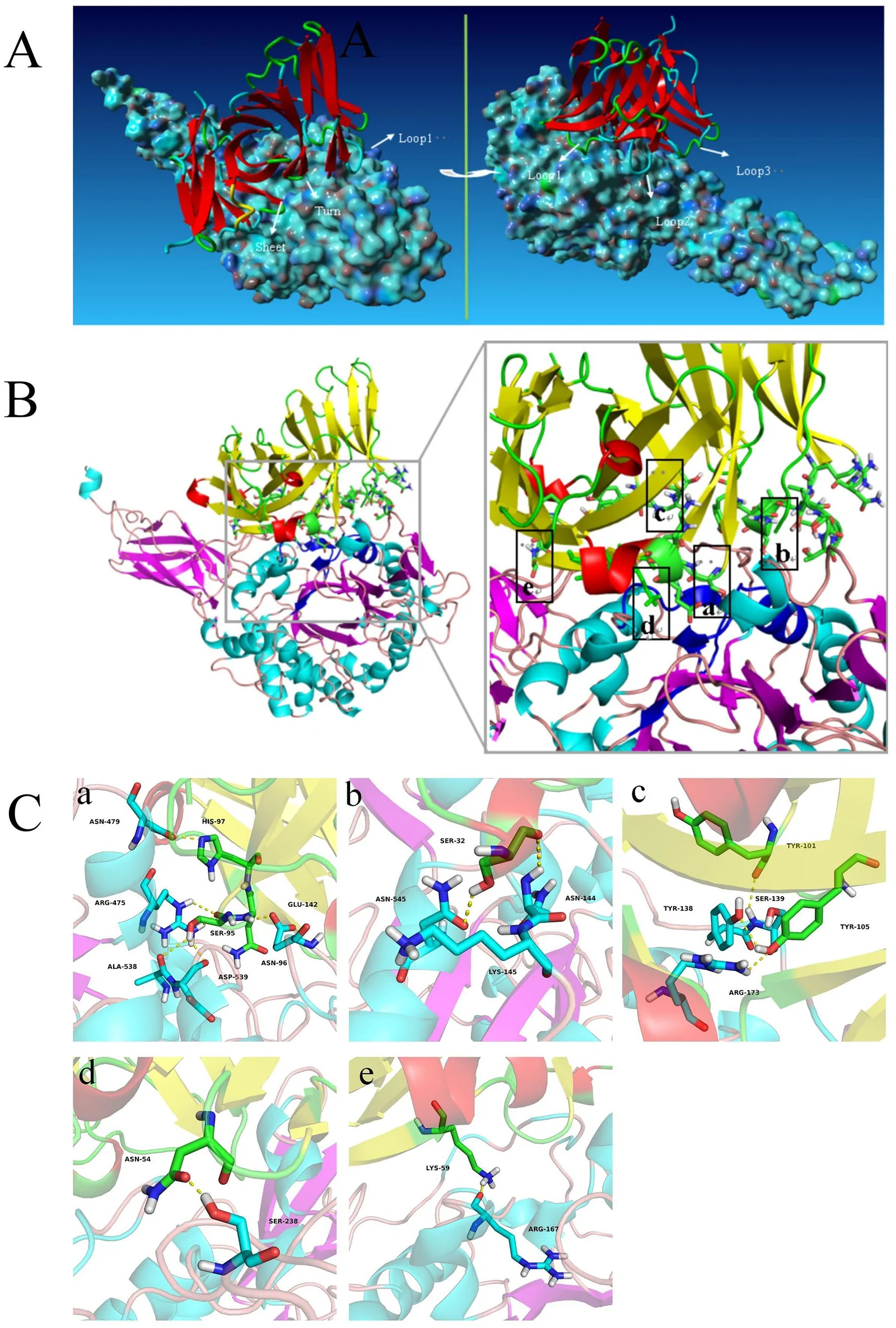
Molecular docking results of the BmChi-IgE complex. **(A)** The overall docked configuration graph of BmChi-IgE complex: BmChi is shown as a surface diagram, mice IgE-Fv segment is shown as a cartoon diagram; **(B)** detailed binding interface of the complex: BmChi in cyan/violet/brown and IgE-Fv in yellow/red/green, with the five principal binding regions (a–e) highlighted in black; **(C)** hydrogen-bond interaction of five main binding regions (a–e). Docking was performed by rigid-body ZDOCK screening followed by RDOCK energy refinement; the displayed pose is the top RDOCK-rescored hit.

To make the molecular determinants of the BmChi-IgE-Fv interaction explicit, the main interfacial residues were extracted from the final docking pose (**Figures 5B, C**) and corroborated by the independent 20 ns MDs interface analysis. On BmChi, the IgE-Fv Turn inserts into the base of the catalytic cavity, which is lined by Asp306, Asp308, Glu310, Trp275, and Trp539, the surrounding concave surface is engaged by Tyr138, Arg167, Arg475, and Asn545, the latter lying within the immunodominant S31 epitope (residues 536-548). On the IgE-Fv, the interaction is mediated by five structural elements, the per-residue hydrogen-bond network for sites a-e is detailed in **Figure 5C**. The final pose was selected as the top RDOCK-rescored hit among ZDOCK poses exceeding a score of 12, the pose combining the highest binding-energy ranking with the strongest shape complementarity and the fewest steric clashes, ensuring the modeled complex is both energetically favourable and biologically consistent with the *in vitro* epitope mapping.

### 3.5 Analysis of molecular dynamics simulations

The structural stability of the complex was continuously monitored using the root-mean-square deviation (RMSD) of the backbone atoms relative to the initial conformation. As depicted in **Supplementary Figure S2**, the complex reached structural equilibrium rapidly after 4 ns. Over the subsequent 16 ns, the RMSD trajectory remained remarkably stable, indicating no significant structural divergence from the initial state (**Supplementary Figure S3**). This stability is further corroborated by the constant center-of-mass distance of the BmChi-IgE complex (**Supplementary Figure S4**) and the stabilized intermolecular binding energy (**Supplementary Figure S5**). Both parameters consistently demonstrate that the complex remained tightly associated without any tendency for dissociation, consistent with the highly specific IgE cross-recognition often observed in arthropod allergens (44). As the results satisfy standard convergence criteria, SMDS provides an orthogonal, non-equilibrium probe of mechanical resistance that does not require long equilibrium sampling. Extending to 100 ns would mainly refine free-energy estimates and is noted as future work.

The dynamic flexibility of specific structural regions was assessed using the root-mean-square fluctuation (RMSF). The antigenic epitope loops of BmChi (Loop 1: Asp127-Lys153; Loop 2: Val160-Leu179; Loop 3: Glu535-Asn540) exhibited minimal structural fluctuations. Correspondingly, key interacting residues on the BmChi epitope (Tyr138, Arg167, Arg475, and Asn545) and the complementary loops of the IgE fragment maintained remarkably low RMSF values (< 1 Å) (**Supplementary Figure S6**). The secondary structures of the BmChi-IgE complex remained fully preserved (**Supplementary Figure S7**), with no solvent-induced disruption observed. This further confirms the robust structural stability of the complex throughout the kinetic process. Collectively, these MD parameters indicate a highly stable, tightly bound the BmChi-IgE complex *in silico*, providing a structural correlate of the IgE-binding activity observed experimentally in the peptide-ELISA screen (2), the simulations describe binding stability, not clinical allergenic potency.

SMDs was employed to map the mechanical unbinding pathway and interaction strength, as it applies continuous external pulling forces to ligands, enabling the observation of dynamic dissociation events that exceed the time scales of traditional equilibrium MDs (21). A 250 ps SMDs was performed by pulling the IgE-Fv away from BmChi along the center-of-mass axis, with representative snapshots extracted at 0, 48, 96, 144, 192, and 240 ps (**Figure 6**). In the initial phase (0–120 ps), the complex demonstrated remarkable resistance to dissociation, evidenced by negligible variations in the mass distance (**Supplementary Figure S8**) alongside a sharp decrease in intermolecular binding energy (**Supplementary Figure S9**). This intense interfacial stability is consistent with the stable protein-IgE binding also observed in chitinase panallergens (3), it reflects persistent complex formation but does not by itself indicate that BmChi is a clinically more potent allergen. This phenomenon indicates that the structural engagement at the epitope-paratope interface is intensely stable, which reflects the constrained surface plasticity and high structural integrity required for potent allergen-antibody complexes. As the simulation progressed beyond 120 ps, the applied force eventually surmounted the binding affinity barriers, leading to conspicuous dissociation and a proportional increase in mass distance. Crucially, throughout this forced unbinding, the intrinsic secondary structures of both proteins remained fully intact (**Supplementary Figure S10**). Concurrently, the progressive interfacial separation led to a steady increase in the solvent-accessible surface area (SASA), which plateaued upon complete dissociation (**Supplementary Figure S11**). This remarkable resistance to mechanical and solvent-driven denaturation mathematically substantiates the exceptional conformational resilience of BmChi under physical stress (12).

**Figure 6:**
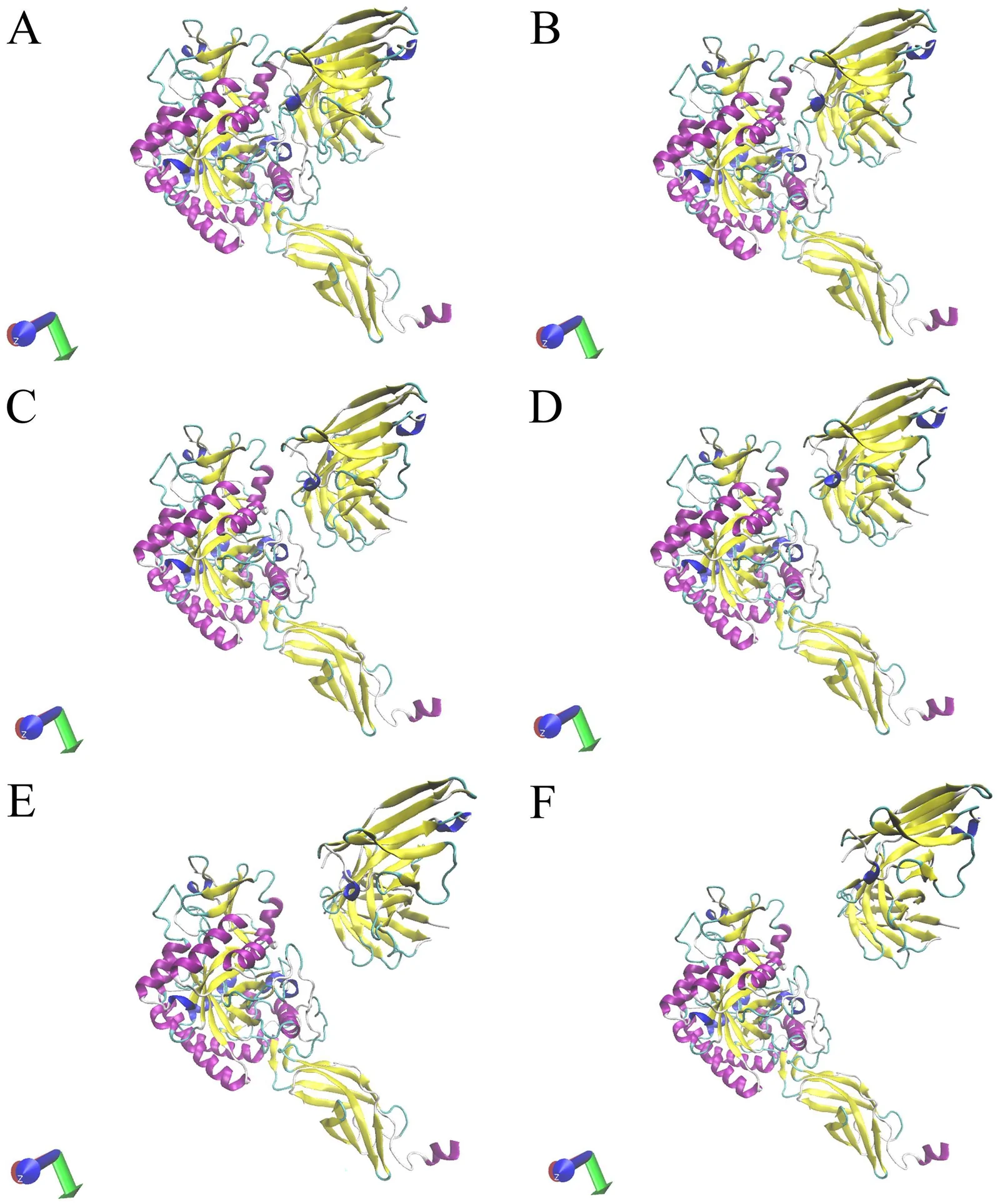
Conformational changes of BmChi-IgE complex during a 250 ps steered molecular dynamics simulation. Snapshots were extracted at **(A)** 0 ps, **(B)** 48 ps, **(C)** 96 ps, **(D)** 144 ps, **(E)** 192 ps, and **(F)** 240 ps, showing progressive interfacial separation under a constant pulling force applied along the center-of-mass axis of the IgE-Fv.

## 4 Discussion

The phylogenetic analysis revealed that BmChi shares significant evolutionary conservation with both insect and plant chitinases. Such conservation across diverse taxa is a recognized feature of panallergens, which are often associated with broad multi-sensitization (3). Given the previously reported cross-reactivity among certain plant chitinase allergens (45), the pronounced phylogenetic proximity observed in this study suggests a possible immunological cross-reactivity between BmChi and taxonomically diverse plant allergens. However, sequence and structural similarity are not, by themselves, a sufficient criterion to establish actual immunological cross-reactivity; several studies also report only limited concordance between *in silico* epitope predictions and experimentally measured IgE binding (46). This possibility must therefore be confirmed experimentally (e.g., by reciprocal IgE-binding inhibition assays and testing with patient-derived sera) before any clinical cross-reactive risk can be inferred.

Consequently, evaluating the clinical cross-reactive risk of emerging insect-derived proteins, such as those from silkworm (*Bombyx mori*), warrants distinct attention in the broader context of food safety (2). As the consumption of insect-based foods continues to expand globally, the potential for cross-reactivity between insect and plant chitinase allergens represents an important consideration for allergen risk assessment and the management of food allergies in sensitized individuals.

While *in silico* prediction efficiently maps theoretical linear epitopes based on primary sequence profiles and secondary structural tendencies, such methodologies inherently possess limitations. Current algorithms predominantly target continuous B-cell epitopes, whereas native, functionally active epitopes on intact allergens are often non-linear and conformationally complex, rendering them highly susceptible to physical and chemical environmental factors (47). To accurately capture these discontinuous conformational epitopes, advanced three-dimensional structural modeling combined with molecular docking simulations is ultimately required to map the spatial interactions between allergens and immune receptors.

Furthermore, despite the high throughput and theoretical accuracy of contemporary predictive software, the inherent assumptions of computational models mandate rigorous experimental validation (48). Therefore, utilizing synthesized peptides representing these computationally predicted regions to assess specific *in vitro* binding capacities with patient-derived IgE antibodies remains essential to conclusively characterize and validate the functional epitopes of BmChi. The integration of computational prediction with experimental peptide mapping, as demonstrated in this study, represents a robust workflow for epitope identification in emerging food allergens.

The identification of the immunodominant linear epitope of BmChi highlights the essential efficacy of combining *in silico* predictions with rigorous *in vitro* peptide mapping. Consistent with recent studies on emerging food allergens, relying solely on computational models can sometimes mischaracterize binding affinities. Thus, experimental validation via overlapping peptides and specific amino acid mutation strategies continues to hold substantial practical importance in research (46, 49, 50).

The robust reactivity of the S31 segment, located near the functionally conserved domain of chitinase, is consistent with recent findings that structurally conserved regions in panallergens can act as immunological triggers, frequently driving cross-reactivity across diverse species (51). These findings not only elucidate the molecular basis of BmChi allergenicity but also provide a vital molecular target for developing hypoallergenic silkworm-derived food products through targeted enzymatic hydrolysis or specific thermal processing techniques aimed at disrupting these epitopes (52). Because such conserved stretches may be shared with homologous allergens, they constitute candidate epitopes that could, in principle, participate in cross-reactivity; whether S31 actually mediates IgE cross-reactivity with plant chitinase allergens remains to be demonstrated experimentally.

Comparison with recent structural epitope studies. The present finding that a single, conserved linear epitope (S31) dominates BmChi IgE recognition is consistent with recent structure-epitope analyses of other food allergens, in which a limited number of immunodominant regions account for most IgE binding (53–55). At the same time, several reports caution that *in silico* epitope predictions and docking scores do not always concord with experimentally measured IgE binding (46), and that cross-reactivity inferred from sequence homology requires verification by inhibition assays (45, 56). We therefore present the BmChi-IgE model as a hypothesis-generating framework to be tested with human allergic sera, rather than as definitive proof of clinical cross-reactivity. Food allergy is a complex type I hypersensitivity in which both protein and carbohydrate determinants can shape IgE responses (57); the present linear-epitope focus therefore represents one facet of a broader immunopathogenesis.

The docking results indicate that the interaction between BmChi and IgE is mediated by a “five-claw” configuration, whereby five key structural elements of the IgE-Fv fragment collectively engage the BmChi surface. This extensive engagement reflects the inherent plasticity and hypervariability of the antibody’s complementarity-determining regions (CDRs), which are essential for the high stability of the BmChi-IgE complex (52). Secondary structure analysis of the binding interface indicates that flexible loops constitute the predominant interacting elements, supplemented by occasional contributions from β-sheets and α-helices. These topological features align precisely with the canonical characteristics of conformational allergenic epitopes. As depicted in the blue-highlighted regions of **Figure 5B**, which coincide with both our preliminary antigenicity assays and computationally predicted epitope regions, strong intermolecular hydrogen bonds are formed involving specific key interfacial residues of BmChi, including Arg173, Ala538, Asn545, and Asp539. These specific electrostatic and hydrogen-bonding networks are predicted to anchor the IgE-Fv to BmChi, supporting the complex formation in the model (58). The robust shape complementarity and extensive hydrogen-bonding network observed in our model are consistent with, and help explain, the IgE-binding activity demonstrated experimentally in the peptide-ELISA screen; they do not by themselves constitute proof of clinical allergenicity. Understanding these specific structural interactions is nevertheless valuable, as modifications to these regions, through specific thermal processing or targeted enzymatic methods, could potentially disrupt the IgE-binding cavity, thereby reducing the allergenic risk of insect-derived alternative proteins (50).

The MDs and SMDs simulations provide insights into the structural basis and stability of the BmChi-IgE interaction, which is consistent with the experimentally observed IgE-binding activity. The low RMSF values observed at the antigen-antibody interface underscore a rigid and stable engagement, a structural hallmark typically associated with highly conserved B-cell epitopes in terrestrial invertebrates (10). The resistance to mechanical unbinding observed in SMD simulations further demonstrates the exceptional conformational resilience of BmChi under physical stress (12).

The structural rigidity and stability observed in the simulations reflect persistent protein-IgE binding, a feature with direct implications for food safety assessments of insect-derived proteins. We emphasize, however, that this stability pertains specifically to the biophysical durability of the BmChi-IgE interaction and does not, in isolation, indicate that BmChi is a clinically more potent allergen. Consequently, mitigating the allergenicity of insect-derived alternative proteins such as BmChi will require targeted interventions designed to irreversibly disrupt these structurally conserved IgE-binding domains and epitopes (9). Future studies should focus on evaluating the efficacy of specific food processing techniques, such as high-temperature treatment (9, 19, 43), Maillard glycation (47), or enzymatic hydrolysis with proteases such as papain or alcalase (9, 43), in disrupting the conformational integrity of BmChi epitopes and reducing IgE reactivity. Ultimately, this study not only bridges the critical knowledge gap regarding BmChi epitopes, but also provides a vital theoretical foundation and structural targets for the future development of precise diagnostics and novel hypoallergenic immunotherapies for edible-insect allergies (59), and the development of analogous allergen-specific immunotherapies guided by epitope mapping (60).

## Data Availability

The original contributions presented in the study are included in the article/**Supplementary Material**. Further inquiries can be directed to the corresponding author.

## Disclaimer of research limitations

This hypothesis-generating study has several limitations that constrain the interpretation of its conclusions, the most significant being the use of mouse-derived anti-BmChi IgE antibodies. Because linear-peptide epitope recognition is mediated by the antibody’s variable (Fv) region, the location of a linear epitope is largely species-independent; in the absence of well-characterized human allergic sera with confirmed clinical reactivity to BmChi, mouse anti-BmChi IgE served as a pragmatic surrogate for mapping IgE-binding sites, and the resulting epitope map therefore identifies candidate linear regions but does not establish their clinical relevance or functional significance, particularly effector-cell activation, in human allergy, so validation with clinically annotated human sera and functional cellular assays remains essential in future studies. In addition, the linear overlapping-peptide ELISA captured only linear epitopes and missed the conformational sites that dominate native IgE binding; the BmChi-IgE model, its hydrogen-bond network, and the dynamic/mechanical stability are computational and await experimental confirmation; the single 20 ns MD run was shorter than the 100 ns typical and lacked independent replicates; no functional or clinical validation was performed; cross-reactivity with plant chitinase allergens was inferred from sequence similarity rather than demonstrated by inhibition assays; and potential selection/confounding biases (single protein source, single antiserum batch, *in vitro*-only design) are declared transparently.

## Supplementary material

Scheme 1 Workflow for BmChi epitope mapping and IgE-binding analysis.

## References

[1] van Huis A . Potential of insects as food and feed in assuring food security. Annu Rev Entomol. (2013) 58:563–83. doi: 10.1146/annurev-ento-120811-153704 23020616

[2] Marzoli F Antonelli P Saviane A Tassoni L Cappellozza S Belluco S . *Bombyx mori* from a food safety perspective: A systematic review. Food Res Int. (2022) 160:111679. doi: 10.1016/j.foodres.2022.111679 36076388

[3] Ribeiro JC Cunha LM Sousa-Pinto B Fonseca J . Allergic risks of consuming edible insects: A systematic review. Mol Nutr Food Res. (2018) 62:1700030. doi: 10.1002/mnfr.201700030 28654197

[4] Sicherer SH Sampson HA . Food allergy: A review and update on epidemiology, pathogenesis, diagnosis, prevention, and management. J Allergy Clin Immunol. (2018) 141:41–58. doi: 10.1016/j.jaci.2017.11.003 29157945

[5] Romero A . Edible insects and allergy risks: Implications for children and the elderly. Allergies. (2025) 5:15. doi: 10.3390/allergies502001530654563

[6] De Marchi L Wangorsch A Zoccatelli G . Allergens from edible insects: Cross-reactivity and effects of processing. Curr Allergy Asthma Rep. (2021) 21:35. doi: 10.1007/s11882-021-01012-z 34056688 PMC8165055

[7] Valenta R Hochwallner H Linhart B Pahr S . Food allergies: the basics. Gastroenterology. (2015) 148:1120–31. doi: 10.1053/j.gastro.2015.02.006 25680669 PMC4414527

[8] Jeong KY Lee JS Yuk JE Song H Lee HJ Kim KJ . Allergenic characterization of Bomb m 4, a 30-kDa Bombyx *mori* lipoprotein 6 from silkworm pupa. Clin Exp Allergy. (2022) 52:888–97. doi: 10.1111/cea.14095 35028994

[9] He W He K Sun F Mu L Liao S Li Q . Effect of heat, enzymatic hydrolysis and acid-alkali treatment on the allergenicity of silkworm pupa protein extract. Food Chem. (2021) 343:128461. doi: 10.1016/j.foodchem.2020.128461 33131957

[10] Nugraha R Kamath SD Johnston E Karnaneedi S Ruethers T Lopata AL . Conservation analysis of B-cell allergen epitopes to predict clinical cross-reactivity between shellfish and inhalant invertebrate allergens. Front Immunol. (2019) 10:2676. doi: 10.3389/fimmu.2019.02676 31803189 PMC6877653

[11] Bannon GA . What makes a food protein an allergen? Curr Allergy Asthma Rep. (2004) 4:43–6. doi: 10.1007/s11882-004-0042-0 14680621

[12] Zhou X Ren L Zhang Y Zhang J Li X Yang A . Effect of structural targeted modifications on the potential allergenicity of peanut allergen Ara h 2. J Agric Food Chem. (2023) 71:836–45. doi: 10.1021/acs.jafc.2c06359 36574959

[13] Yue W Huang S Lin S Feng X Yan L Yang Z . Prediction, identification, and analysis of major B-cell linear epitopes of bomb m 6 from silkworm pupa. Food Chem. (2025) 463:141211. doi: 10.1016/j.foodchem.2024.141211 39305666

[14] Catalán A García C Sambra V Cadena N Rojas J Arán-Sekul T . Predictive analysis of B-cell antigenic epitopes in phospholipase D toxins from *Loxosceles* spiders. Toxicon X. (2025) 26:100222. doi: 10.1016/j.toxcx.2025.100222 40230574 PMC11994341

[15] Leoni C Volpicella M Dileo M Gattulli B Ceci LR . Chitinases as food allergens. Molecules. (2019) 24:2087. doi: 10.3390/molecules24112087 31159327 PMC6600546

[16] Sánchez-Monge R Blanco C Perales AD Collada C Carrillo T Aragoncillo C . Class I chitinases, the panallergens responsible for the latex-fruit syndrome, are induced by ethylene treatment and inactivated by heating. J Allergy Clin Immunol. (2000) 106:190–5. doi: 10.1067/mai.2000.107599 10887324

[17] Zhao X Li L Kuang Z Luo G Li B . Proteomic and immunological identification of two new allergens from silkworm (*Bombyx mori* L.) pupae. Cent Eur J Immunol. (2015) 40:30–4. doi: 10.5114/ceji.2015.50830 26155181 PMC4472537

[18] Pomés A Mueller GA Chruszcz M . Structural aspects of the allergen-antibody interaction. Front Immunol. (2020) 11:2067. doi: 10.3389/fimmu.2020.02067 32983155 PMC7492603

[19] Verhoeckx KCM Vissers YM Baumert JL Faludi R Feys M Flanagan S . Food processing and allergenicity. Food Chem Toxicol. (2015) 80:223–40. doi: 10.1016/j.fct.2015.03.005 25778347

[20] Fernández-Quintero ML Loeffler JR Waibl F Kamenik AS Hofer F Liedl KR . Conformational selection of allergen-antibody complexes-surface plasticity of paratopes and epitopes. Protein Eng Des Sel. (2019) 32:513–23. doi: 10.1093/protein/gzaa014 32719844 PMC7451023

[21] Gómez-Velasco H García-Ramírez B Siliqi D Graewert MA Quintero-Martinez A Ortega E . Allergen-induced structural rearrangements in IgE: insights from SAXS and molecular dynamics. Int J Biol Macromol. (2025) 328:147658. doi: 10.1016/j.ijbiomac.2025.147658 40953619

[22] Masum MHU Mahdeen AA Barua L Parvin R Heema HP Ferdous J . Developing a chimeric multiepitope vaccine against Nipah virus (NiV) through immunoinformatics, molecular docking and dynamic simulation approaches. Microb Pathog. (2024) 197:107098. doi: 10.1016/j.micpath.2024.107098 39521154

[23] Zhao J Zeng J Liu Y Lin H Gao X Wang H . Understanding the mechanism of increased IgG/IgE reactivity but eecreased immunodetection recovery in thermally induced shrimp (*Litopenaeus vannamei*) tropomyosin via multispectroscopic and molecular dynamics simulation techniques. J Agric Food Chem. (2023) 71:3444–58. doi: 10.1021/acs.jafc.2c08221 36750428

[24] Cong Y Li L . Identification of the critical amino acid residues of immunoglobulin E and immunoglobulin G epitopes in β-lactoglobulin by alanine scanning analysis. J Dairy Sci. (2012) 95:6307–12. doi: 10.3168/jds.2012-5543 22939793

[25] Dahiya N Tewari R Hoondal GS . Biotechnological aspects of chitinolytic enzymes: A review. Appl Microbiol Biotechnol. (2006) 71:773–82. doi: 10.1007/s00253-005-0183-7 16249876

[26] Altschul SF Wootton JC Gertz EM Agarwala R Morgulis A Schaffer AA . Protein database searches using compositionally adjusted substitution matrices. FEBS J. (2005) 272:5101–9. doi: 10.1111/j.1742-4658.2005.04945.x 16218944 PMC1343503

[27] Altschul SF Koonin EV . Iterated profile searches with PSI-BLAST--a tool for discovery in protein databases. Trends Biochem Sci. (1998) 23:444–7. doi: 10.1016/S0968-0004(98)01298-5 9852764

[28] Goodman RE Ebisawa M Ferreira F Sampson HA van Ree R Vieths S . AllergenOnline: a peer-reviewed, curated allergen database to assess novel food proteins for potential cross-reactivity. Mol Nutr Food Res. (2016) 60:1183–98. doi: 10.1002/mnfr.201500769 26887584

[29] Mari A Scala E Palazzo P Ridolfi S Zennaro D Carabella G . Bioinformatics applied to allergy: allergen databases, from collecting sequence information to data integration. The Allergome platform as a model. Cell Immunol. (2007) 244:97–100. doi: 10.1016/j.cellimm.2007.02.012 17434469

[30] Lv Z Ji F Song J Li P Chen M Chang J . Predicting the spatial structure of membrane protein and B-cell epitopes of the MPXV_VEROE6 strain of monkeypox virus. Heliyon. (2023) 9:e20386. doi: 10.1016/j.heliyon.2023.e20386 37767496 PMC10520823

[31] Jespersen MC Peters B Nielsen M Marcatili P . BepiPred-2.0: improving sequence-based B-cell epitope prediction using conformational epitopes. Nucleic Acids Res. (2017) 45:W24–9. doi: 10.1093/nar/gkx346 28472356 PMC5570230

[32] Sui ZY Wan MM Liu Q Lv XQ Liu JY Zhao YH . Identification of IgE epitopes and analysis of critical amino acid residues in fish parvalbumin. J Agric Food Chem. (2025) 73:21578–91. doi: 10.1021/acs.jafc.5c06323 40814803

[33] Yang F Zhang H Wang S Zhao X Tang Y Zeng J . Peptidomics and molecular docking reveal digestion-resistant IgE-binding epitopes in bovine β-lactoglobulin and α-lactalbumin from simulated infant digestion. Food Chem. (2025) 493:145829. doi: 10.1016/j.foodchem.2025.145829 40782637

[34] Sharma N Patiyal S Dhall A Pande A Arora C Raghava GPS . AlgPred 2.0: an improved method for predicting allergenic proteins and mapping of IgE epitopes. Brief Bioinform. (2021) 22:bbaa294. doi: 10.1093/bib/bbaa294 33201237

[35] Chen R Li L Weng Z . ZDOCK: an initial-stage protein-docking algorithm. Proteins. (2003) 52:80–7. doi: 10.1002/prot.10389 12784371

[36] Li L Chen R Weng Z . RDOCK: refinement of rigid-body protein docking predictions using soft-repulsive potentials. Proteins. (2003) 53:693–707. doi: 10.1002/prot.10460 14579360

[37] Alem M Abtahi Froushani SM Hajighahramani N Hosseini-Asl S Pourfarzi F Nemati R . Immuno-informatics voyage through molecular mimicry of Heat Shock Proteins: Potential IBD immunopathogenesis. PloS One. (2025) 20:e0333618. doi: 10.1371/journal.pone.0333618 41100508 PMC12530574

[38] Jumper J Evans R Pritzel A Green T Figurnov M Ronneberger O . Highly accurate protein structure prediction with AlphaFold. Nature. (2021) 596:583–9. doi: 10.1038/s41586-021-03819-2 34265844 PMC8371605

[39] Kwon H Ko S Ha K Lee JK Choi Y . Assessing the predictive ability of computational epitope prediction methods on Fel d 1 and other allergens. PloS One. (2024) 19:e0306254. doi: 10.1371/journal.pone.0306254 39178274 PMC11343462

[40] Ullah H Mahmud S Hossain MJ Islam MSB Kibria KMK . Immunoinformatic identification of the epitope-based vaccine candidates from Maltoporin, FepA and OmpW of *Shigella* Spp, with molecular docking confirmation. Infect Genet Evol. (2021) 96:105129. doi: 10.1016/j.meegid.2021.105129 34737105

[41] Colovos C Yeates TO . Verification of protein structures: Patterns of nonbonded atomic interactions. Protein Sci. (1993) 2:1511–9. doi: 10.1002/pro.5560020916 8401235 PMC2142462

[42] Lu Q Xie H Qu M Liu T Yang Q . Group h chitinase: A molecular target for the development of lepidopteran-specific insecticides. J Agric Food Chem. (2023) 71:5944–52. doi: 10.1021/acs.jafc.2c08845 37038745

[43] Xu F Zhao Y Han Z Zhang X Chen B Zhu X . Sustainable emerging proteins: Allergenic proteins in edible insects, microalgae, and microorganisms, and desensitization processing technologies. Foods. (2025) 15:69. doi: 10.3390/foods15010069 41517134 PMC12785758

[44] Pali-Schöll I Meinlschmidt P Larenas-Linnemann D Purschke B Hofstetter G Rodríguez-Monroy FA . Edible insects: Cross-recognition of IgE from crustacean- and house dust mite allergic patients, and reduction of allergenicity by food processing. World Allergy Organ J. (2019) 12:100006. doi: 10.1016/j.waojou.2018.10.001 30937131 PMC6439408

[45] Zhang Y Che H Li C Jin T . Food allergens of plant origin. Foods. (2023) 12:2232. doi: 10.3390/foods12112232 37297475 PMC10252803

[46] McClain S . Bioinformatic screening and detection of allergen cross-reactive IgE-binding epitopes. Mol Nutr Food Res. (2017) 61:1600676. doi: 10.1002/mnfr.201600676 28191711 PMC5573986

[47] Deng J Yang X Shen W Gan C Cao J Pan D . Cold plasma-promoted glycation of bovine caseins: glycoproteomic insights into conformational relaxation and epitope masking. J Agric Food Chem. (2026) 74:8916–32. doi: 10.1021/acs.jafc.5c18007 41804691

[48] Ma X Wang F Yu N Zhang J Wang X Xie M . Homology modeling of sesame allergenic protein and prediction of B-cell linear antigenic epitopes using immunoinformatic tools. Foods. (2025) 14:4158. doi: 10.3390/foods14234158 41376094 PMC12692619

[49] Hu W Xie F Wu Y Meng X Yang A Wu Z . Glutamine and isoleucine as the key amino acids in major IgE linear epitopes of α-Lactalbumin identified by the immune peptide microarray assay for Chinese bovine milk allergy. J Agric Food Chem. (2025) 73:15346–55. doi: 10.1021/acs.jafc.5c04676 40462490

[50] Sundararaj R Mathimaran A Prabhu D Ramachandran B Jeyaraman J Muthupandian S . *In silico* approaches for the identification of potential allergens among hypothetical proteins from *Alternaria alternata* and its functional annotation. Sci Rep. (2024) 14:6696. doi: 10.1038/s41598-024-55463-1 38509156 PMC10954717

[51] Khammeethong T Phiriyangkul P Khumsui S Meeboongirt P Suriyabhivadh NA Yokthongwattana C . Serological cross-reactivity and potential allergenic risk of edible cricket proteins in children and adolescents with house dust mite allergy. J Agric Food Chem. (2026) 74:9735–48. doi: 10.1021/acs.jafc.5c12201 41840881 PMC13022853

[52] Günal-Köroğlu D Karabulut G Ozkan G Yılmaz H Gültekin-Subaşı B Capanoglu E . Allergenicity of alternative proteins: Reduction mechanisms and processing strategies. J Agric Food Chem. (2025) 73:7522–46. doi: 10.1021/acs.jafc.5c00948 40105205 PMC11969658

[53] Chen Y He X Yang S Huan F Li D Yang Y . IgE epitope analysis and hypo-immunoreactivity derivative of arginine kinase in mantis shrimp (*Oratosquilla oratoria*). J Agric Food Chem. (2023) 71:9508–18. doi: 10.1021/acs.jafc.3c01549 37289596

[54] He X Yang Y Chen Y Kang S Li F Li D . Immunoglobulin E epitope mapping and structure-allergenicity relationship analysis of crab allergen Scy p 9. J Agric Food Chem. (2023) 71:17379–90. doi: 10.1021/acs.jafc.3c04970 37931089

[55] Li Y Sun X Yang L . Structure/epitope analysis and IgE binding activities of three cyclophilin family proteins from *Dermatophagoides pteronyssinus*. Sci Rep. (2023) 13:13630. doi: 10.1038/s41598-023-40720-6 37604978 PMC10442367

[56] Wagner S Breiteneder H . The latex-fruit syndrome. Biochem Soc Trans. (2002) 30:935–40. doi: 10.1042/bst0300935 12440950

[57] Hils M Wolbing F Hilger C Fischer J Hoffard N Biedermann T . The history of carbohydrates in type I allergy. Front Immunol. (2020) 11:586924. doi: 10.3389/fimmu.2020.586924 33163001 PMC7583601

[58] Dhouib W Frikha F Rebai A Kharrat N . DeepPlantAllergy: deep learning for explainable prediction of allergenicity in plant proteins. Brief Bioinform. (2025) 26:bbaf605. doi: 10.1093/bib/bbaf605 41236479 PMC12616846

[59] Trifonova D Curin M Focke-Tejkl M Liu Z Borochova K Fomina D . Recombinant hypoallergenic cat *allergy* vaccines. Allergy. (2025) 80:2622–35. doi: 10.1111/all.16542 40178413 PMC12444911

[60] Keswani T LaHood NA Marini-Rapoport O Karmakar B Andrieux L Reese B . Neutralizing IgG4 antibodies are a biomarker of sustained efficacy after peanut oral immunotherapy. J Allergy Clin Immunol. (2024) 153:1611–1620.e7. doi: 10.1016/j.jaci.2024.02.017 38460677 PMC11894811

