# Supplementary material for: Mapping the major B-cell linear epitope of silkworm (Bombyx mori) pupal chitinase and molecular dynamics insights into its IgE-binding stability
Source: Front Immunol. 2026 Oct 1;17:1942595. doi: 10.3389/fimmu.2026.1942595 (PMC13668528; doi:10.3389/fimmu.2026.1942595)
Supplement: Supplementary file 1 [file SupplementaryFile1.pdf]

Supplementary Material

Mapping the major B-cell linear epitope of silkworm (*Bombyx mori*) pupal chitinase and molecular dynamics insights into its IgE-binding stability

Lei Gu[1,*], Xiangjie Zhao[2], Lei Yan[1], Lin Bo[3], Xuemei Sui[1]

[1] Affiliated Huai'an No. 1 People's Hospital of Nanjing Medical University, Huai'an 223300, Jiangsu, China

[2] Huai'an University, College of Life Science and Food Engineering, Huai'an 223003, Jiangsu, China

[3] The Second Affiliated Hospital of Soochow University, Suzhou 215004, Jiangsu, China

*Corresponding author e-mail: gleiha@163.com (L. Gu)

Supplementary Table S1. Overlapping peptide design for mapping BmChi epitopes

Peptide No.	Peptide sequence (N---C)	Peptide No.	Peptide sequence (N---C)	Peptide No.	Peptide sequence (N---C)
P1	MGHRMIALVFAVAAF	P38	FIPICGGDGINDSLK	P75	KEAQKYMDHIFVMSY
P2	IALVFAVAAFAVGSC	P39	GGDGINDSLKEIEGS	P76	YMDHIFVMSYDFKGA
P3	AVAAFAVGSCGPPGK	P40	NDSLKEIEGSFQALQ	P77	FVMSYDFKGAWSNDT
P4	AVGSCGPPGKPNLGW	P41	EIEGSFQALQRSCSG	P78	DFKGAWSNDTLGHQT
P5	GPPGKPNLGWGERTF	P42	FQALQRSCSGREDFK	P79	WSNDTLGHQTALYAP
P6	PNLGWGERTFAIVEV	P43	RSCSGREDFKVSIHD	P80	LGHQTALYAPSWSPK
P7	GERTFAIVEVNQAAT	P44	REDFKVSIHDPWAAL	P81	ALYAPSWSPKETYTT
P8	AIVEVNQAATAYNQL	P45	VSIHDPWAALQKPQK	P82	SWSPKETYTTDFGIR
P9	NQAATAYNQLVTKKD	P46	PWAALQKPQKGLSSW	P83	ETYTTDFGIRYLLTQ
P10	VTKKDAADVSVNWNV	P47	QKPQKGLSSWNEPYK	P84	DFGIRYLLTQGVQPK
P11	VTKKDAADVSVNWNV	P48	GLSSWNEPYKGNFGQ	P85	YLLTQGVQPKKLVVG
P12	AADVSVNWNVWTGDA	P49	NEPYKGNFGQLMQLK	P86	GVQPKKLVVGVAMYG
P13	VNWNVWTGDAADKSR	P50	GNFGQLMQLKQANTG	P87	KLVVGVAMYGRGWTG
P14	WTGDAADKSRVLLDK	P51	LMQLKQANTGLKVLP	P88	VAMYGRGWTGVHDYN
P15	ADKSRVLLDKKEVWS	P52	QANTGLKVLPSIGGW	P89	RGWTGVHDYNDDIPF
P16	VLLDKKEVWSGAGSA	P53	LKVLPSIGGWTLADP	P90	VHDYNDDIPFTGVAN
P17	KEVWSGAGSATSAAF	P54	SIGGWTLADPFFFFT	P91	DDIPFTGVANGPVKG
P18	GAGSATSAAFKVKKG	P55	TLADPFFFFTDKTKR	P92	TGVANGPVKGTWQDG
P19	TSAAFKVKKGGRYQM	P56	FFFFTDKTKRDRFVA	P93	GPVKGTWQDGVVDYR

P20	KVKKGGRYQMQVELC	P57	DKTKRDRFVASVKDF	P94	TWQDGVVDYREIVNG
P21	GRYQMQVELCNSDGC	P58	DRFVASVKDFLQTWK	P95	VVDYREIVNGITSGT
P22	QVELCNSDGCSSSEG	P59	SVKDFLQTWKFFDGV	P96	EIVNGITSGTWQYFY
P23	NSDGCSSSEGVEIVV	P60	LQTWKFFDGVDIDWE	P97	ITSGTWQYFYDKVAQ
P24	SSSEGVEIVVADTDG	P61	FFDGVDIDWEFPGGK	P98	WQYFYDKVAQAPYVW
P25	VEIVVADTDGSHLRP	P62	DIDWEFPGGKGANPD	P99	DKVAQAPYVWNPTTG
P26	ADTDGSHLRPLDYSI	P63	FPGGKGANPDLGSPK	P100	APYVWNPTTGDLVTY
P27	SHLRPLDYSIGEKNK	P64	GANPDLGSPKDGDVY	P101	NPTTGDLVTYDDARS
P28	LDYSIGEKNKPFKQT	P65	LGSPKDGDVYVQLMK	P102	DLVTYDDARSVIEKG
P29	GEKNKPFKQTSGKVV	P66	DGDVYVQLMKELRQM	P103	DDARSVIEKGKYVRN
P30	PFKQTSGKVVGAYFV	P67	VQLMKELRQMLDELA	P104	VIEKGKYVRNNKLGG
P31	SGKVVGAYFVEWGVY	P68	ELRQMLDELAAETGR	P105	KYVRNNKLGGLFAWE
P32	GAYFVEWGVYPRKFP	P69	LDELAAETGRTYELT	P106	NKLGGLFAWEIDADN
P33	EWGVYPRKFPVDRVP	P70	AETGRTYELTSAISA	P107	LFAWEIDADNGDILN
P34	PRKFPVDRVPVPNLT	P71	TYELTSAISAGWDKI	P108	IDADNGDILNAMNMG
P35	VDRVPVPNLTHLLYG	P72	SAISAGWDKIQVVNY	P109	GDILNAMNMGLGNSA
P36	VPNLTHLLYGFIPIC	P73	GWDKIQVVNYKEAQK		
P37	HLLYGFIPICGGDGI	P74	QVVNYKEAQKYMDHI		

Supplementary Table S2. Overlapping peptides redesign of high IgE reactivity of P24 and P108 peptide from BmChi epitopes

Peptide No.	Sequence redesign	Peptide sequence (N---C)	Peptide No.	Sequence redesign	Peptide sequence (N---C)
S0	**P24**	116SSSEG VEIVV ADTDG130	S00	**P108**	536IDADN GDILN AMNMG550
S1	P24-Δ1N	117SSEG VEIVV ADTDG130	S25	P108-Δ1N	537DADN GDILN AMNMG550
S2	P24-Δ2N	118SEG VEIVV ADTDG130	S26	P108-Δ2N	538ADN GDILN AMNMG550
S3	P24-Δ3N	119EG VEIVV ADTDG130	S27	P108-Δ3N	539DN GDILN AMNMG550
S4	P24-Δ4N	120G VEIVV ADTDG130	S28	P108-Δ4N	540N GDILN AMNMG550
S5	P24-Δ5N	121VEIVV ADTDG130	S29	P108-Δ5N	541GDILN AMNMG550
S6	P24-Δ1C	116SSSEG VEIVV ADTD129	S30	P108-Δ1C	536IDADN GDILN AMNM549
S7	P24-Δ2C	116SSSEG VEIVV ADT128	S31	P108-Δ2C	536IDADN GDILN AMN548
S8	P24-Δ3C	116SSSEG VEIVV AD127	S32	P108-Δ3C	536IDADN GDILN AM547
S9	P24-Δ4C	116SSSEG VEIVV A126	S33	P108-Δ4C	536IDADN GDILN A546
S10	P24-Δ5C	116SSSEG VEIVV125	S34	P108-Δ5C	536IDADN GDILN545
S11	S116A	116ASSEG VEIVV ADTDG130	S35	I536A	536ADADN GDILN AMNMG550
S12	S117A	116SASEG VEIVV ADTDG130	S36	D537A	536IAADN GDILN AMNMG550
S13	S118A	116SSAEG VEIVV ADTDG130	S37	D539A	536IDAAN GDILN AMNMG550
S14	E119A	116SSSAG VEIVV ADTDG130	S38	N540A	536IDADA GDILN AMNMG550
S15	G120A	116SSSEA VEIVV ADTDG130	S39	G541A	536IDADN ADILN AMNMG550
S16	V121A	116SSSEG AEIVV ADTDG130	S40	D542A	536IDADN GAILN AMNMG550
S17	E122A	116SSSEG VAIVV ADTDG130	S41	I543A	536IDADN GDALN AMNMG550

S18	I123A	116SSSEG VEAVV ADTDG130	S42	L544A	536IDADN GDIAN AMNMG550
S19	V124A	116SSSEG VEIAV ADTDG130	S43	N545A	536IDADN GDILA AMNMG550
S20	V125A	116SSSEG VEIVA ADTDG130	S44	M547A	536IDADN GDILN AANMG550
S21	D127A	116SSSEG VEIVV AATDG130	S45	N548A	536IDADN GDILN AMAMG550
S22	T128A	116SSSEG VEIVV ADADG130	S46	M549A	536IDADN GDILN AMNAG550
S23	D129A	116SSSEG VEIVV ADTAG130	S47	G550A	536IDADN GDILN AMNMA550
S24	G130A	116SSSEG VEIVV ADTDA130			

Supplementary Table S3. Sequence alignment results of BmChi with high homology chitinase templates

PDB No.	Title	Score	Query coverage	Similarity	Identity
1RD6	Chitinase A	811	0.99	0.52	0.69
2WK2	Chitinase A	809	0.96	0.53	0.72
2WLY	Chitinase A	808	0.96	0.53	0.72
1FFR	Chitinase A	803	0.96	0.53	0.72
1NH6	Chitinase A	803	0.96	0.53	0.72
1CTN	Chitinase A	803	0.96	0.53	0.72
1EHN	Chitinase A	803	0.96	0.53	0.72
1FFQ	Chitinase A	802	0.96	0.52	0.72

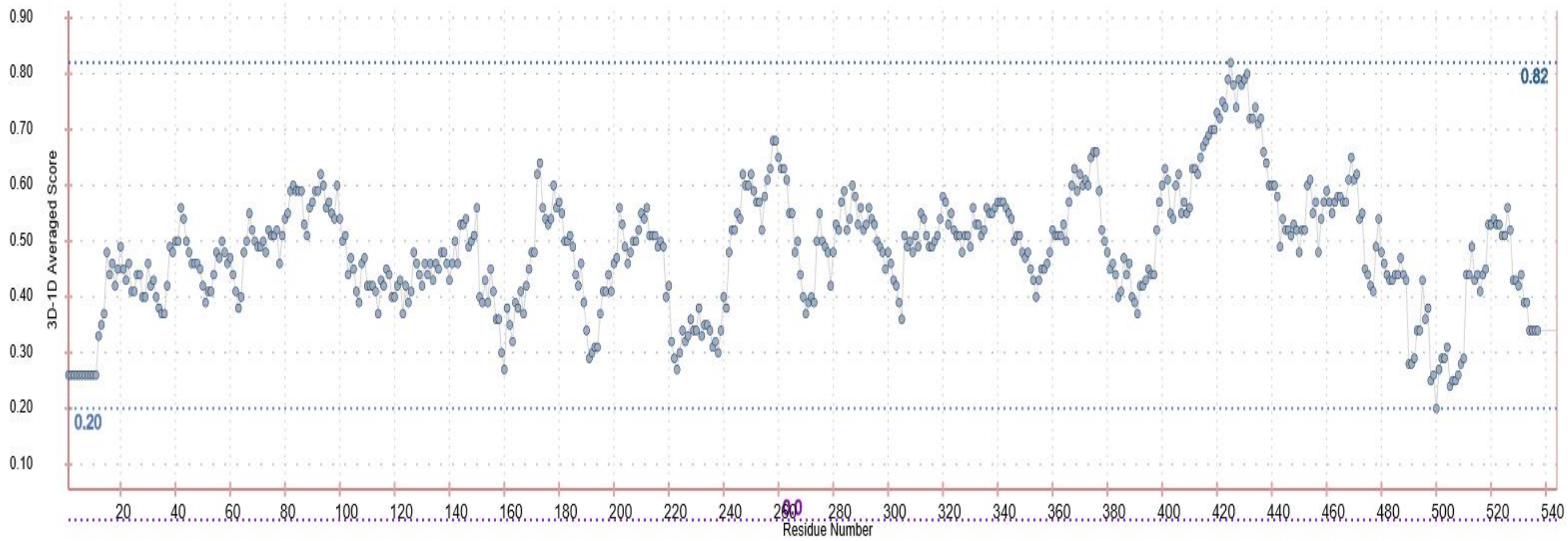

Supplementary Figure S1. Verify 3D results plot of the modelling BmChi amino acid residues

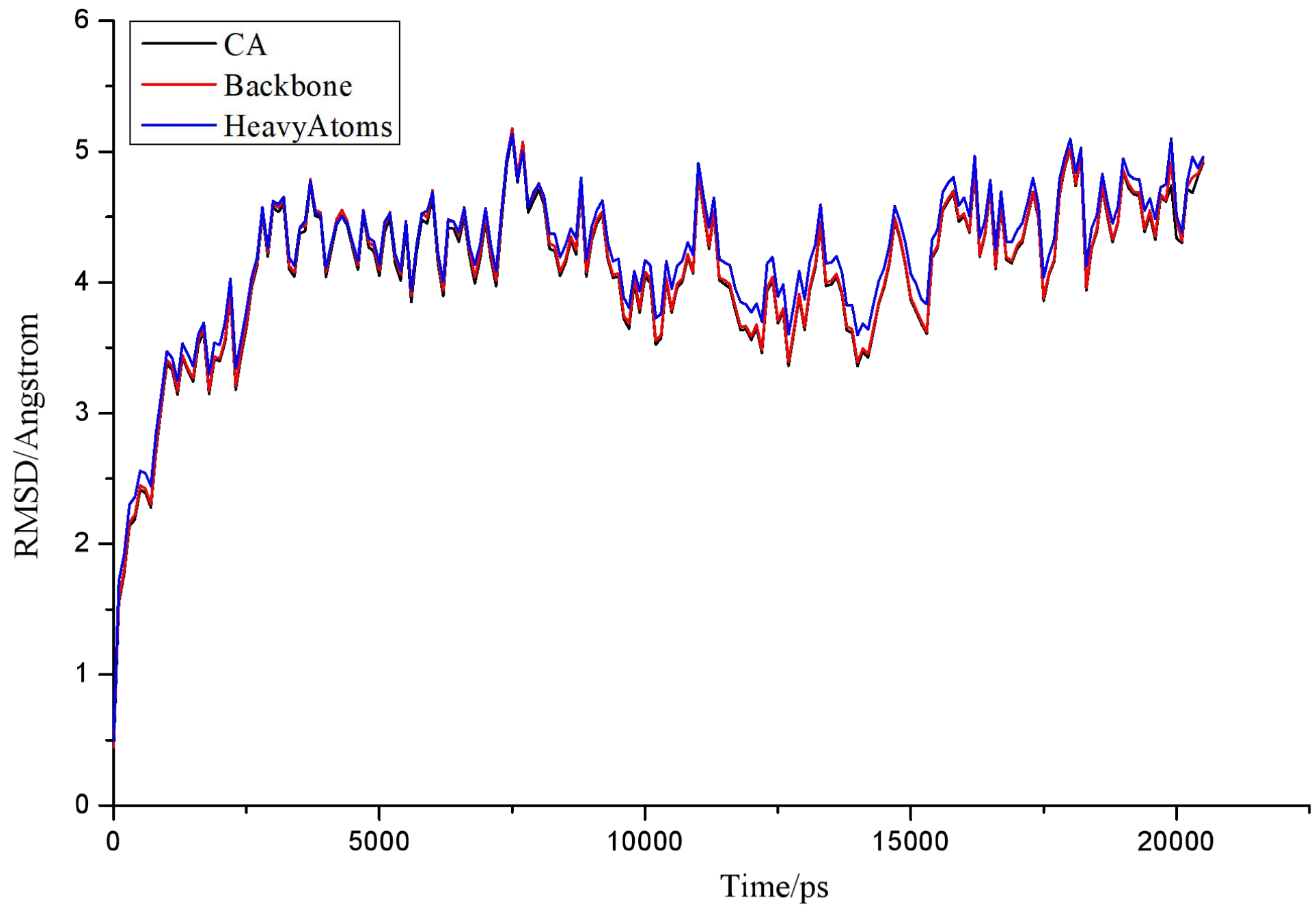

Supplementary Figure S2. RMSD analysis of different atoms in molecular simulation time

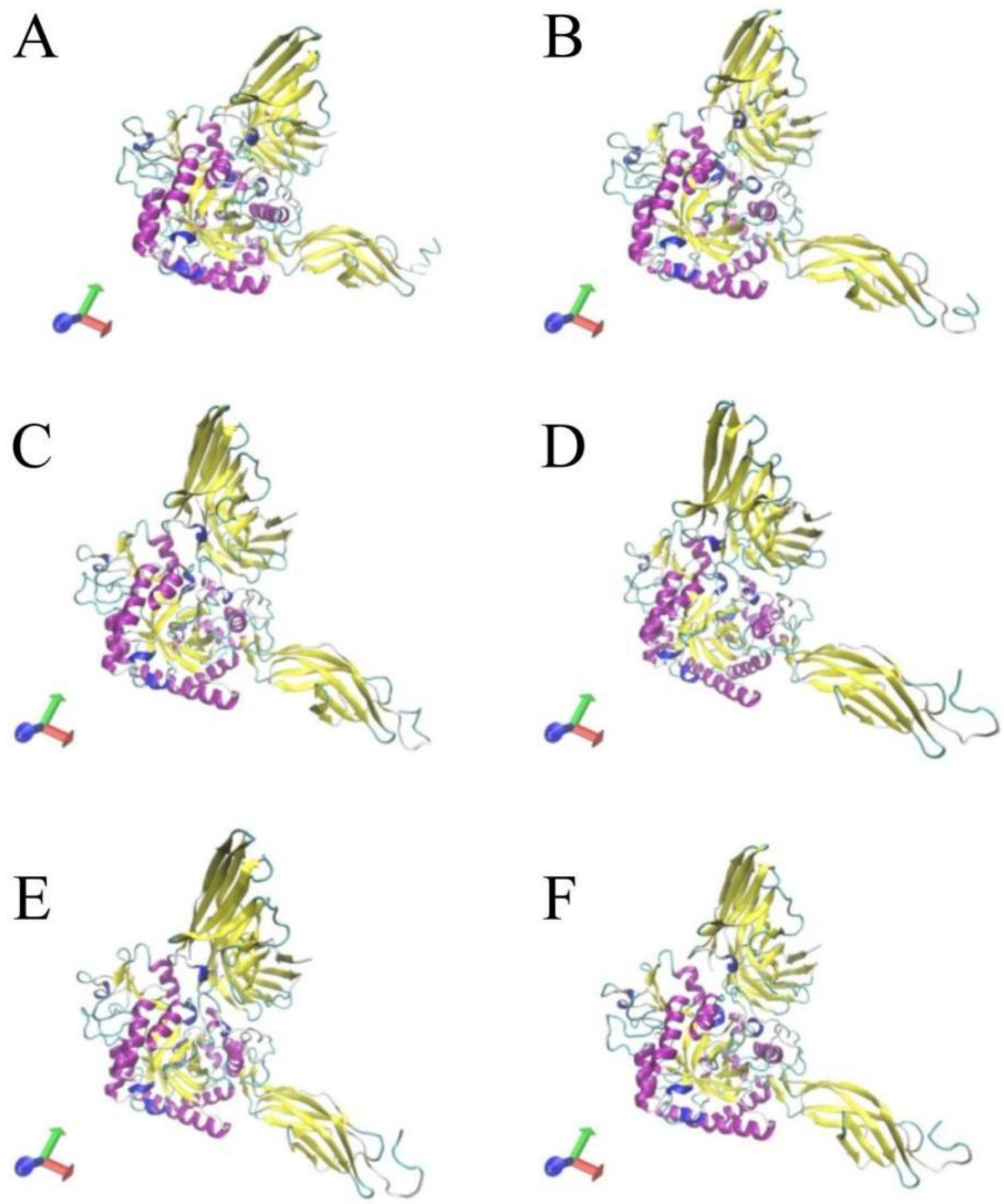

Supplementary Figure S3. Conformational changes of BmChi-IgE complex in different molecular dynamic simulation time (A. 0 ns; B. 4 ns; C. 8 ns; D. 12 ns; E. 16 ns; F. 20 ns)

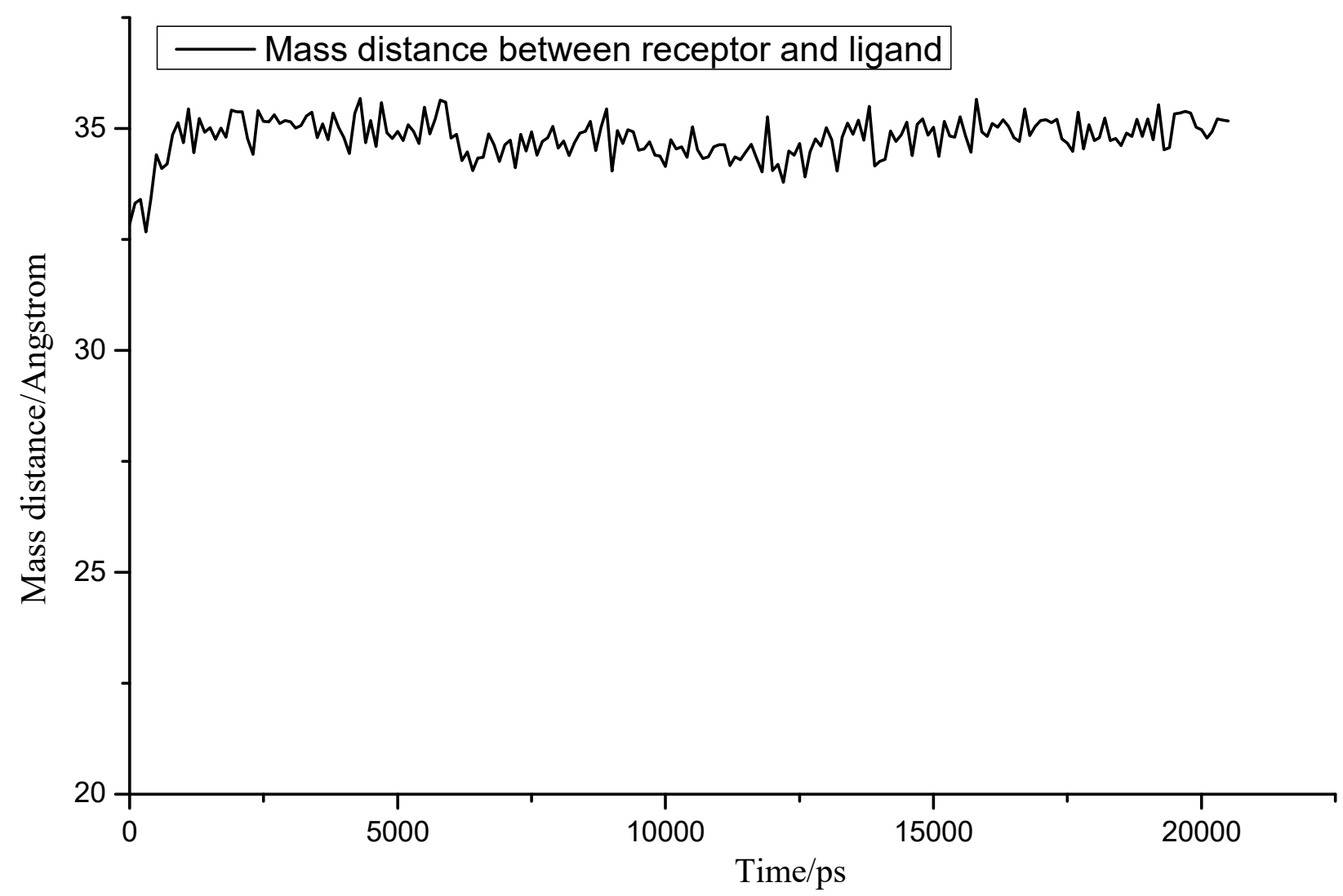

•

Supplementary Figure S4. Mass distance of BmChi-IgE complex in molecular dynamic simulation time

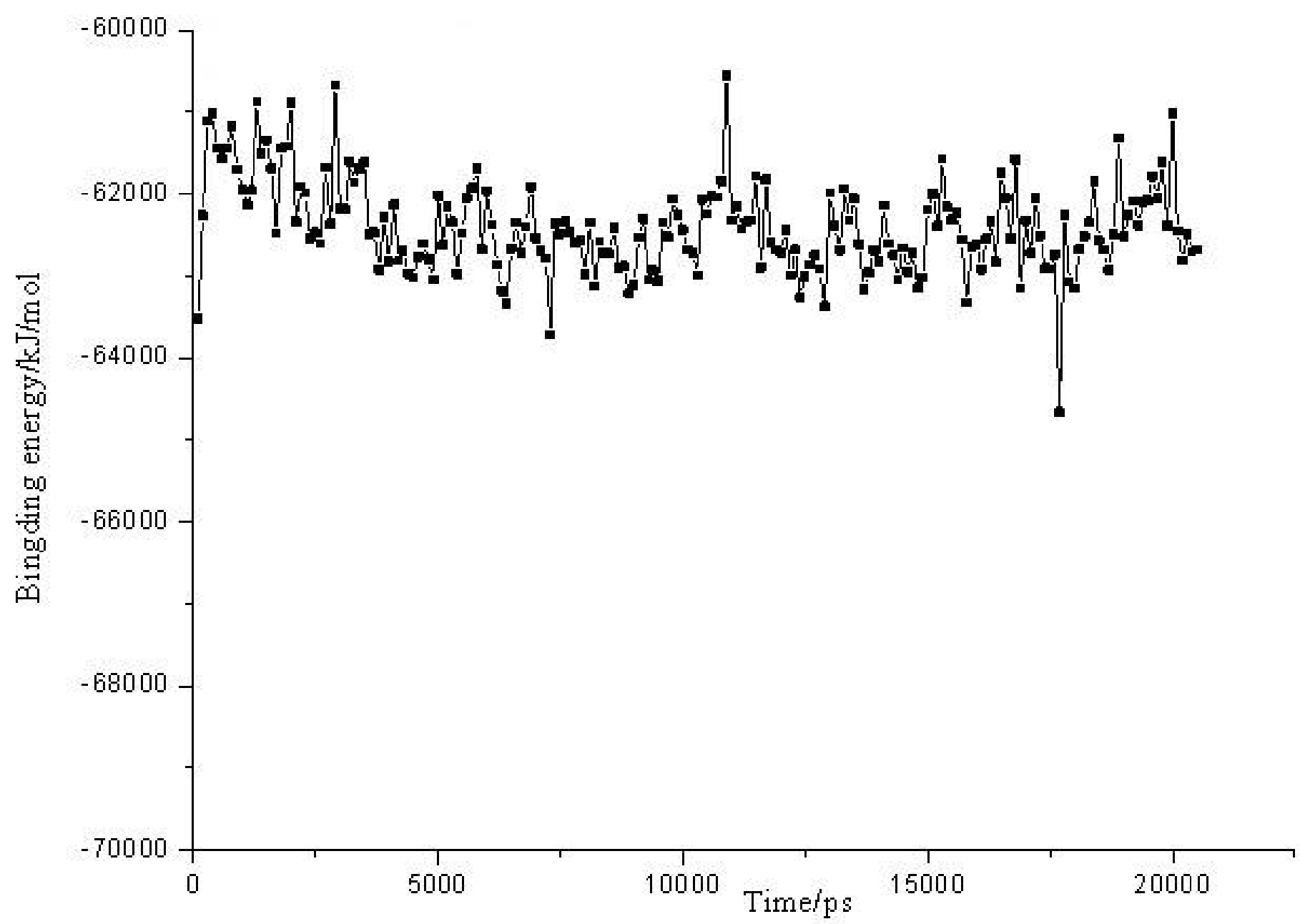

Supplementary Figure S5. Binding energy changes of BmChi-IgE complex in molecular dynamic simulation

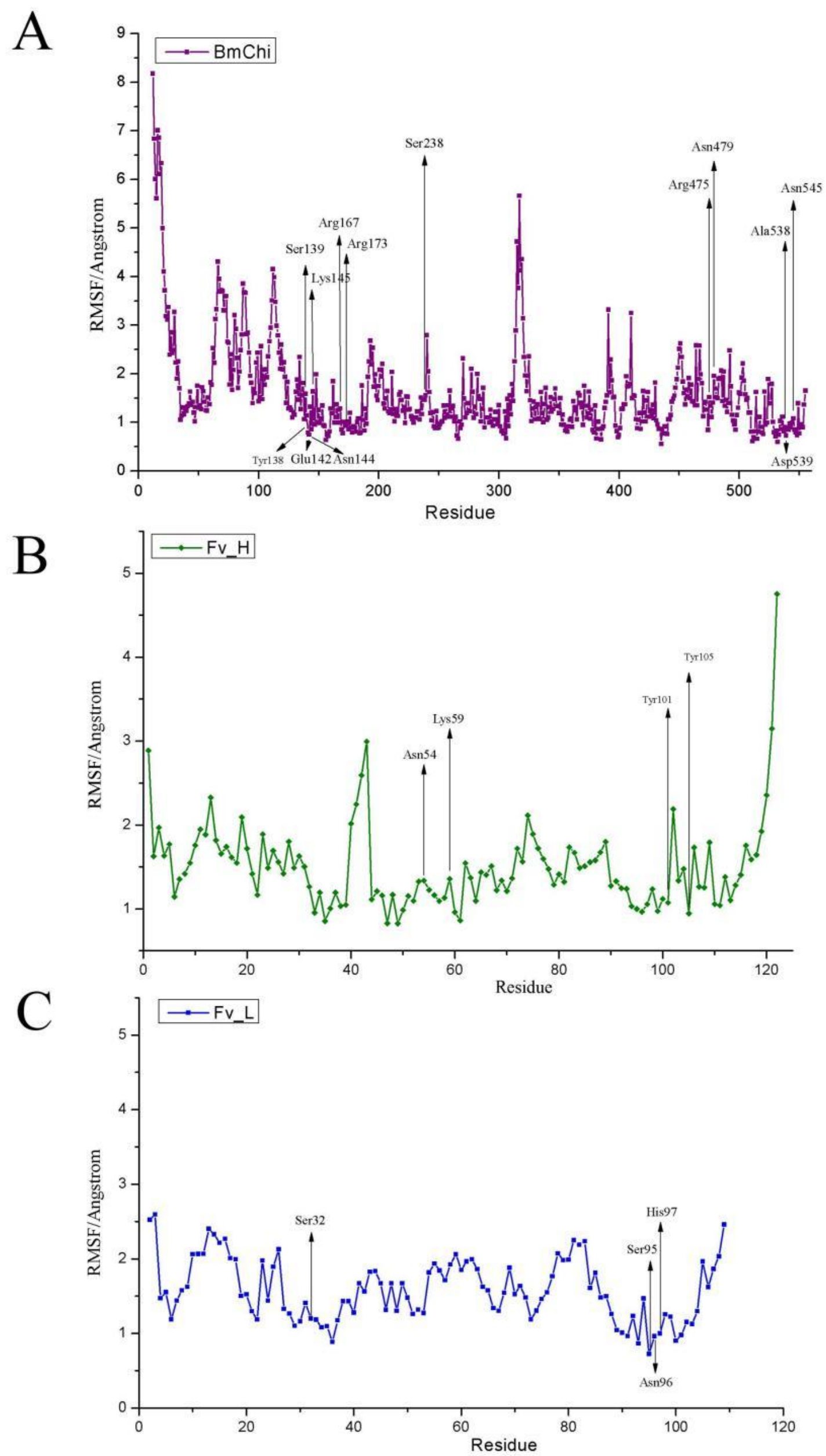

Supplementary Figure S6. RMSF analysis of amino acid residues of BmChi (A), heavy chain (B) and light chain (C) of IgE in molecular dynamic simulation

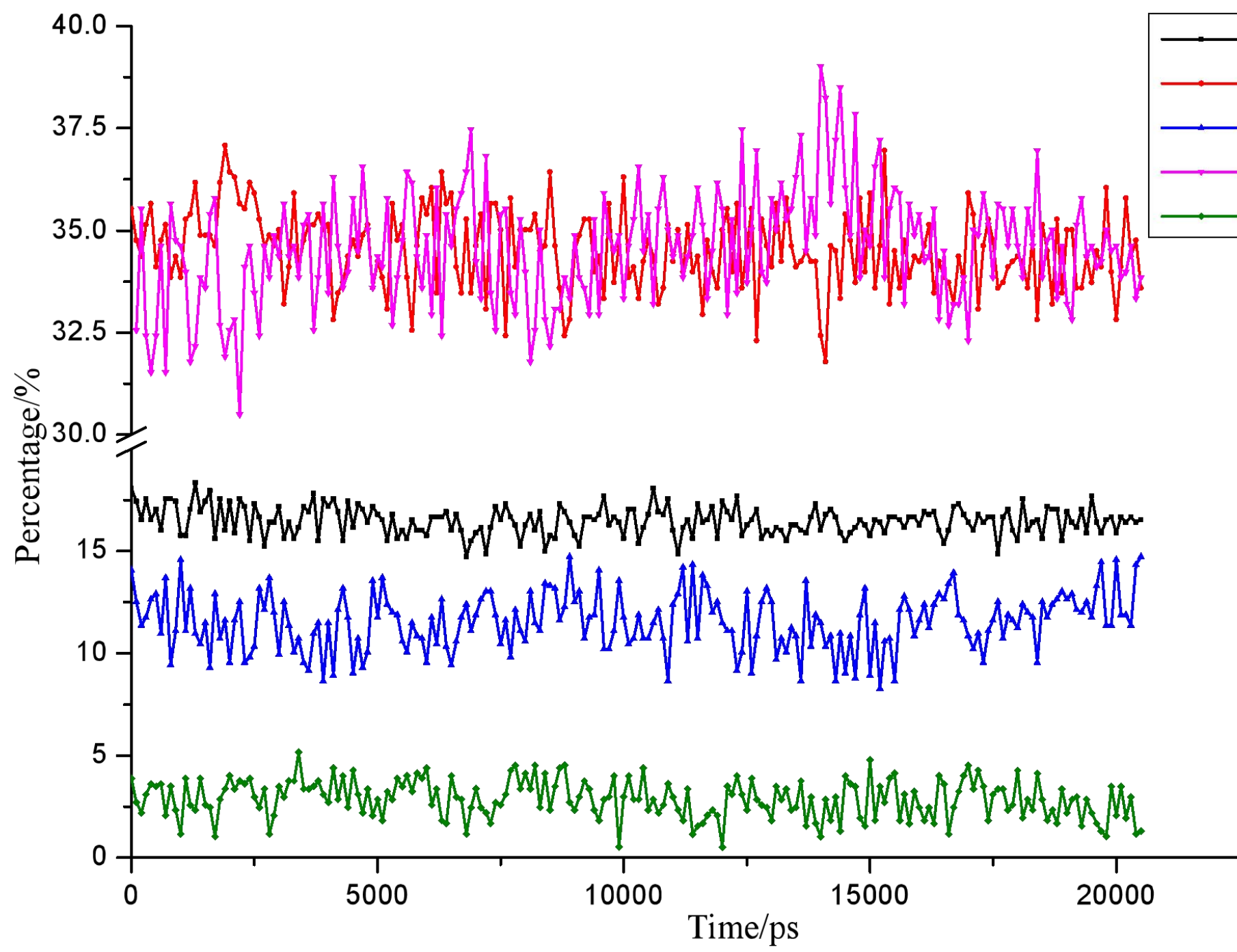

Supplementary Figure S7. Secondary structure changes of BmChi-IgE complex in molecular dynamic simulation

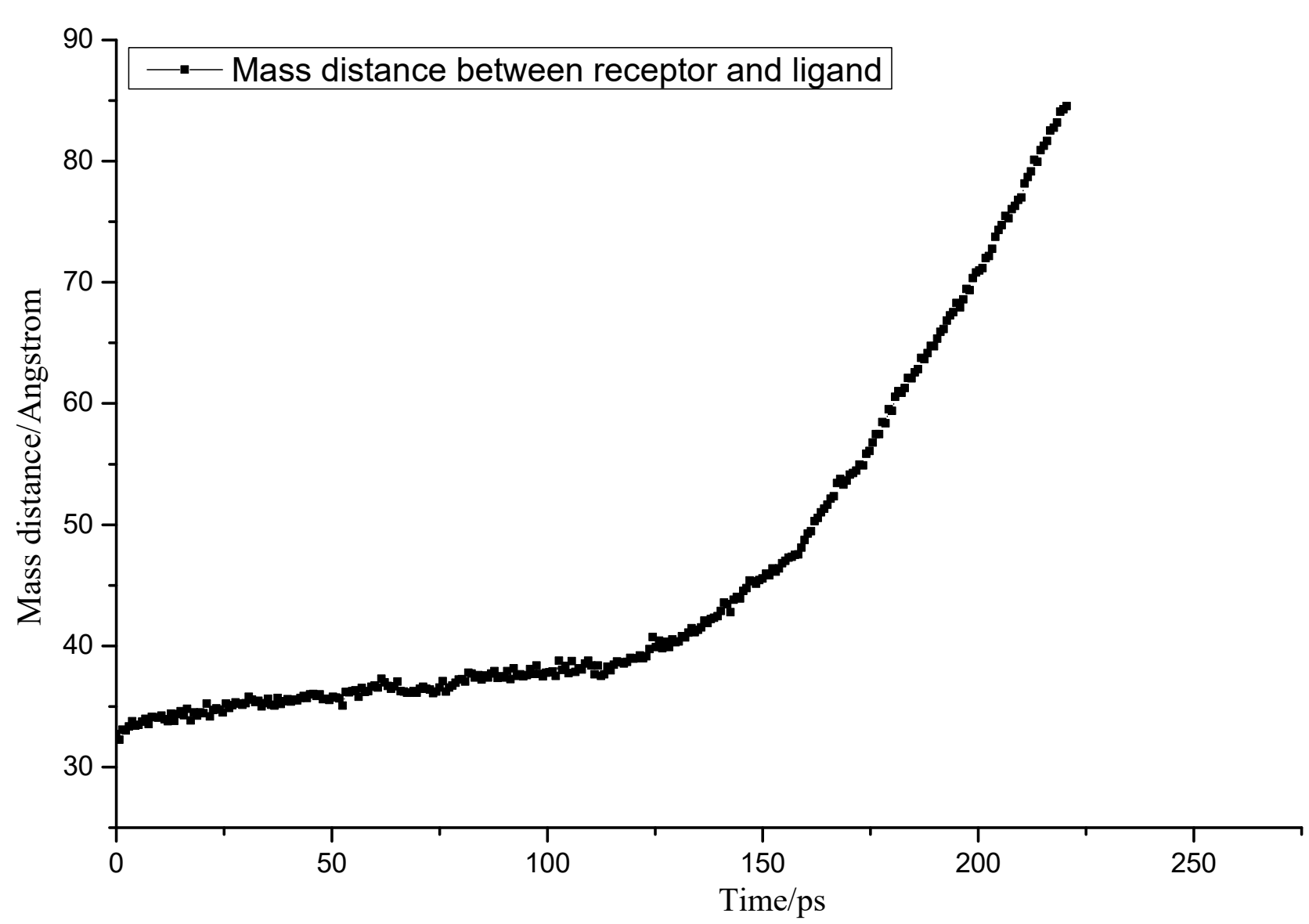

Supplementary Figure S8. Mass distance of BmChi-IgE complex in steered molecular dynamics simulation time

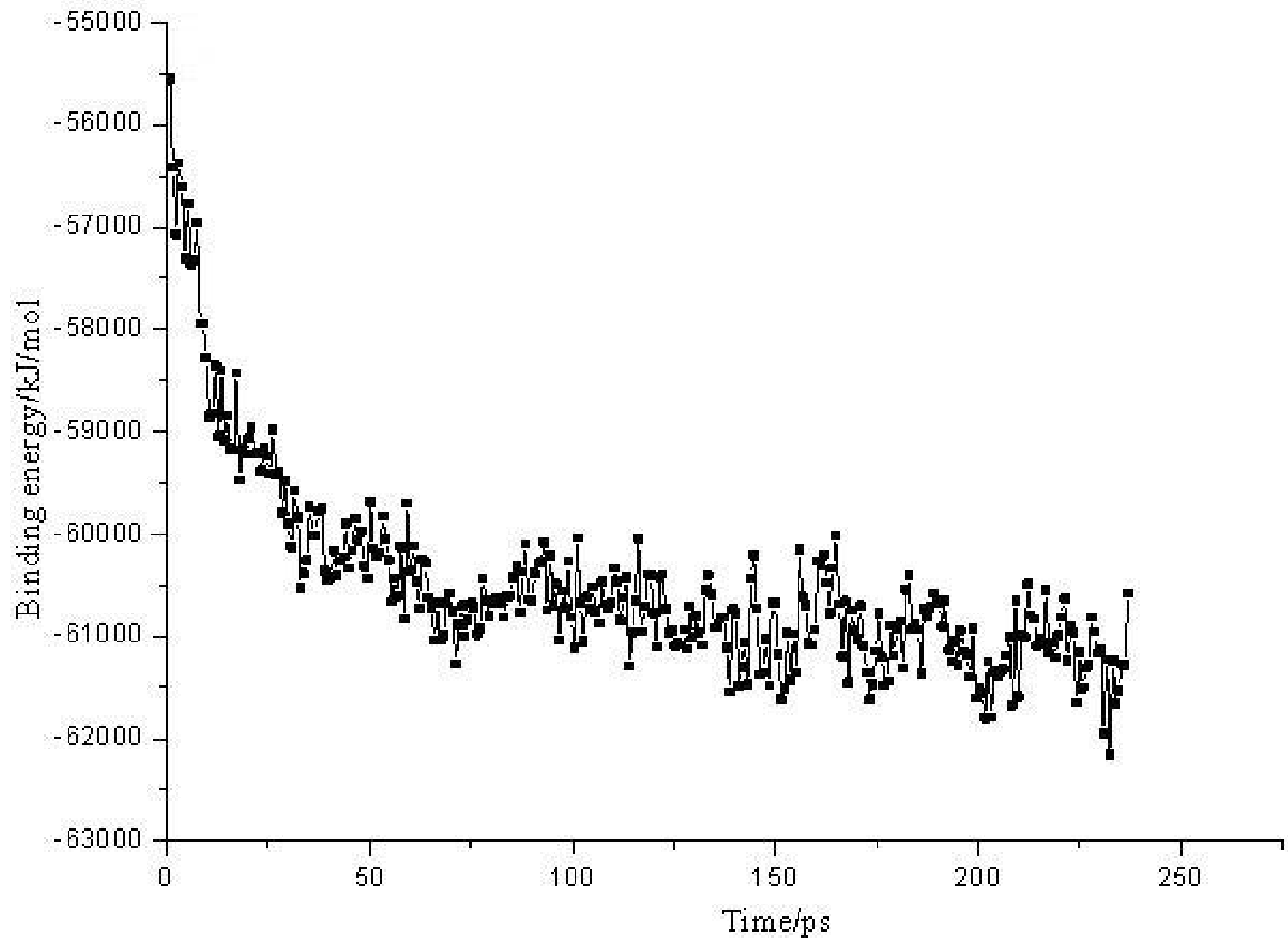

Supplementary Figure S9. Binding energy changes of BmChi-IgE complex in steered molecular dynamics simulation time

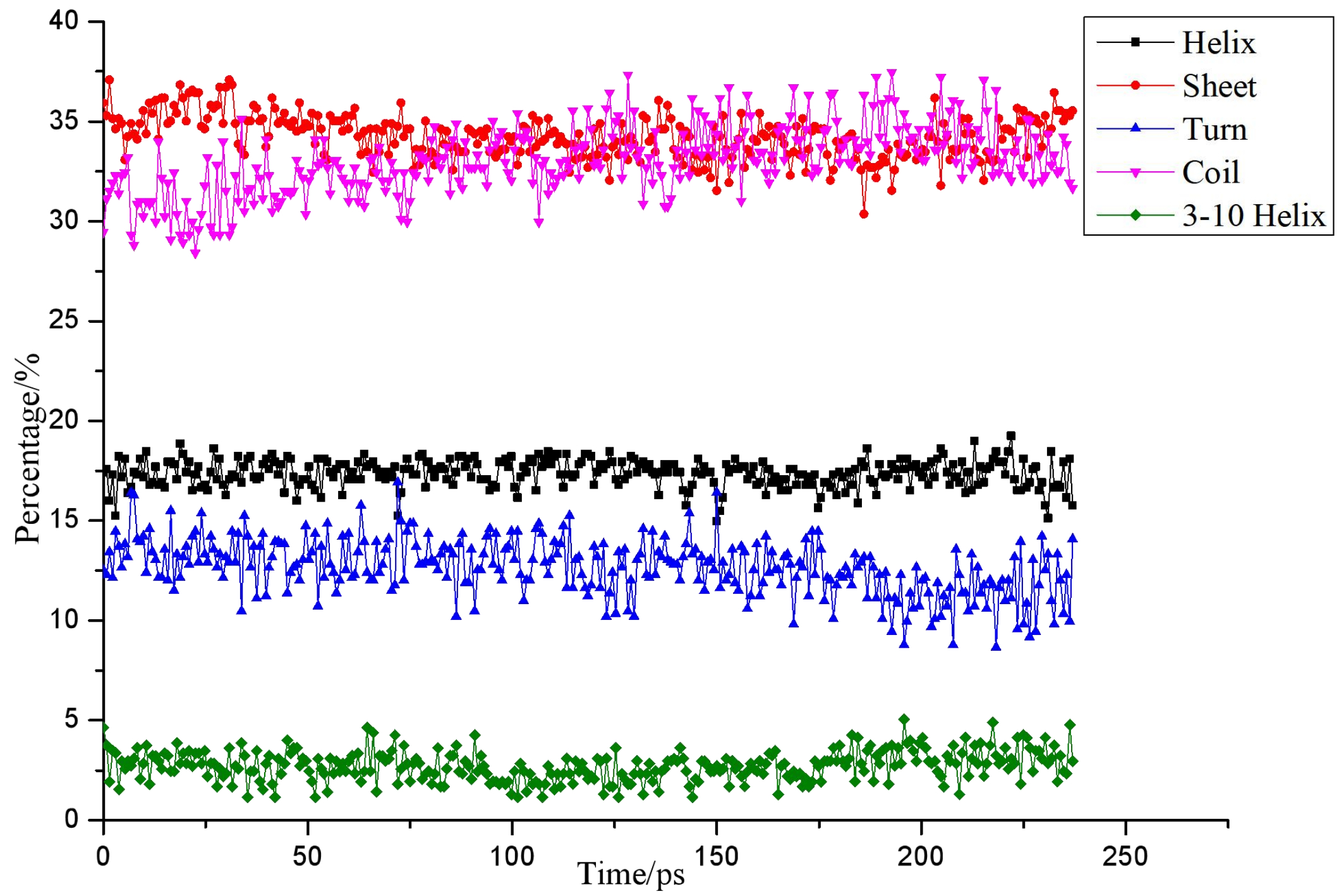

Supplementary Figure S10. Secondary structure changes of BmChi-IgE complex in steered molecular dynamics simulation time

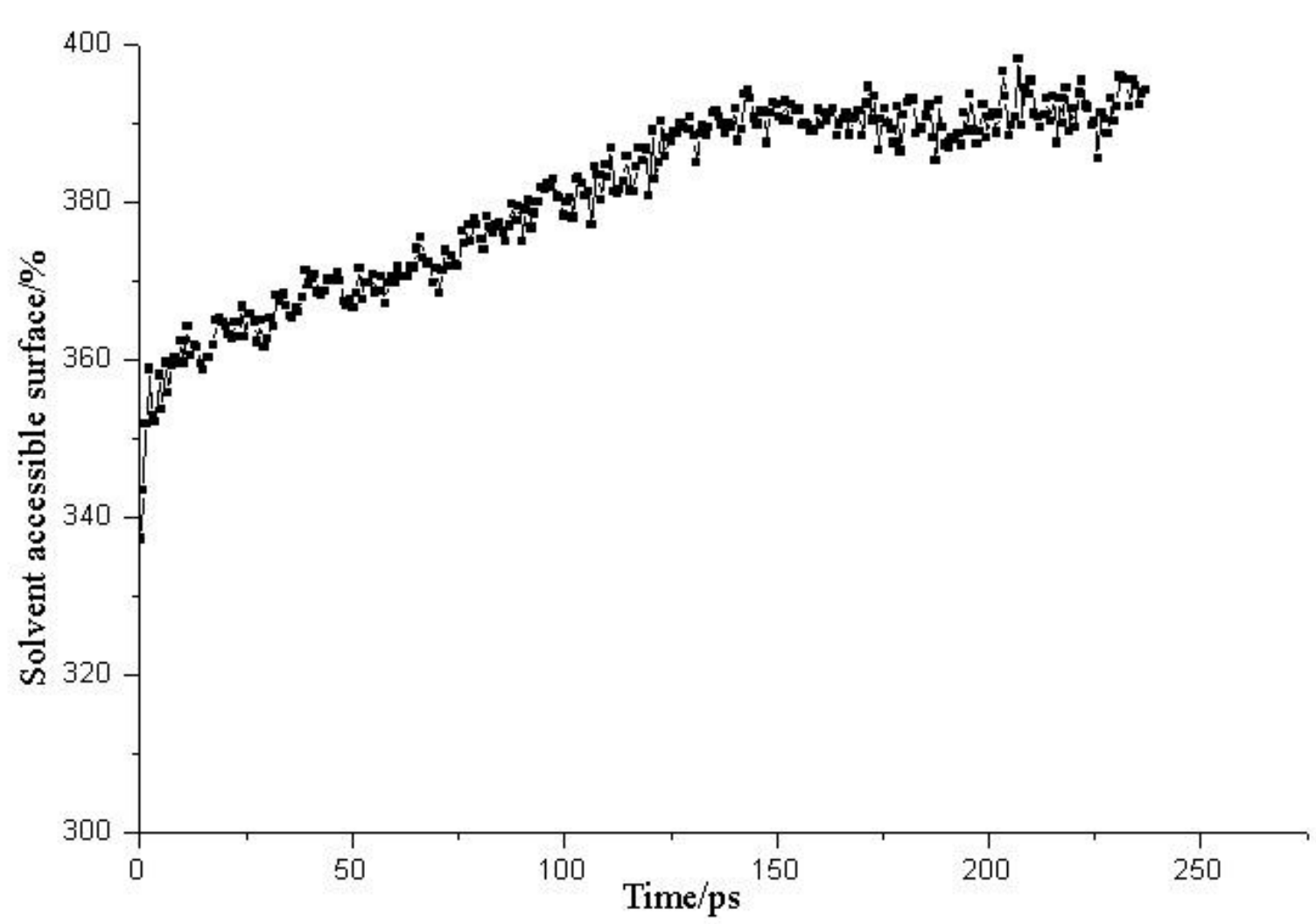

Supplementary Figure S11. Solvent accessible surface area of BmChi-IgE complex in steered molecular dynamics simulation time

Supplementary Methods

1. Gene cloning and expression

The target gene (1668 bp) was cloned into the pMD19 vector and introduced into *E. coli* JM109 competent cells via heat shock transformation. Positive transformants were identified by blue-white screening on LB agar plates supplemented with ampicillin (100 µg/mL), and plasmid DNA was isolated from confirmed clones. The target fragment was subsequently subcloned into the pCold I expression vector using double restriction enzyme digestion and ligation. Recombinant plasmids were verified by colony PCR and restriction analysis, and the correct construct, designated CRF841-2, was transformed into *E. coli* BL21 T1R competent cells. Positive transformants were selected and subjected to protein expression induction.

2. Strain culture and protein induction

A single colony of the CRF841-2 transformed BL21 T1R strain was inoculated into 2 mL of LB medium containing ampicillin (100 µg/mL) and cultured overnight at 37°C with shaking (220 rpm). For protein expression, 100 µL of this pre-culture was transferred into 5 mL of fresh LB medium supplemented with ampicillin (100 µg/mL) in a sterile glass tube. The culture was incubated at 37°C with shaking until the optical density at 600 nm (OD_{600}) reached ~0.6. The culture was then cooled statically at 15°C for 15 min, followed by addition of 50 µL of 100 mM IPTG (final concentration: 1 mM) to induce recombinant protein expression. Induction was carried out at 15°C for 22 h with gentle shaking (120 rpm).

3. Protein extraction and Western Blot analysis

Bacterial cells were harvested by centrifugation (4,000 × g, 10 min, 4°C), and the pellet corresponding to 2.0 OD units was resuspended in 320 µL of ice-cold PBS (pH 7.4). Cells were lysed by sonication on ice (3 × 15 s pulses, 30% amplitude, with 30 s intervals), and the lysate was clarified by centrifugation (12,000 × g, 5 min, 4°C). The supernatant (soluble fraction) and pellet (insoluble fraction) were collected separately, and the total lysate (uncentrifuged) was retained as the "total protein" sample. For SDS-PAGE analysis, 8 µL of each fraction (equivalent to 0.05 OD units) was mixed with 2 µL of 5× SDS loading buffer, denatured at 95°C for 10 min, and loaded onto a 12.5% polyacrylamide gel. Electrophoresis was performed at a constant current of 25 mA per gel for ~70 min. Gels were stained with Coomassie Brilliant Blue R-250 (CBB-R250) and destained using standard destaining solution.

Protein expression was confirmed by Western blot analysis. For each protein fraction, namely, total lysate, supernatant, and pellet, 8 µL (equivalent to 0.05 OD units) was mixed with 2 µL of 5× SDS loading buffer, denatured at 95 °C for 10 min, and resolved by SDS-PAGE using both a prestained protein molecular weight marker and a His-tag-specific marker. Electrophoresis was carried out at a constant current of 25 mA per gel for approximately 80 min on a 12.5% polyacrylamide gel (Figure S12). The PVDF membrane and filter papers were cut to match the dimensions of the gel, pre-wetted in transfer buffer, and assembled in the following order between the electrode plates of the electrophoretic transfer apparatus: filter paper, PVDF membrane, gel, filter paper. Electroblotting was performed at a constant current of 36 mA for 80 min.

The transferred PVDF membrane was blocked by incubation at 37 °C for 1 h in 10 mL of blocking buffer (1.5% w/v BSA in TBS). Subsequently, the membrane was incubated with 5 mL of diluted Penta-His antibody (primary antibody) for 1 h at room temperature. After one wash with 20 mL of TBST buffer (Tris-buffered saline containing 0.05% Tween-20) and two brief rinses with TBS buffer, the membrane was incubated with 5 mL of diluted horseradish peroxidase (HRP)–conjugated rabbit anti-mouse IgG antibody (secondary antibody) for 1 h. The membrane was then washed once with 20 mL of TBST buffer and rinsed twice with TBS buffer. Finally, colorimetric detection was performed using 1 mL of TrueBlue™ Peroxidase Substrate for 1 min. The resulting immunoblot is presented in Figure S13.
